# Placental iron transport under maternal stress: a missing link in foetal programming and mental health

**DOI:** 10.1016/j.ebiom.2026.106170

**Published:** 2026-02-13

**Authors:** Mariana Schroeder, Nan Yi, Barbara Fuenzalida, Timothee C. Furrer, Therina du Toit, Martin Mueller, Edgar Ontsouka, Christiane Albrecht

**Affiliations:** a Faculty of Medicine, Institute of Biochemistry and Molecular Medicine, University of Bern, Switzerland; b Department of Biomedical Research, University of Bern, Switzerland; c Division of Gynecology and Obstetrics, Lindenhofgruppe, Bern, Switzerland

**Keywords:** Placental adaptations, Iron transfer, Prenatal stress, Sex differences

## Abstract

**Background:**

Environmental stress and iron deficiency are increasingly recognised as prevalent challenges during pregnancy, with significant implications for both maternal and foetal health. Environmental stressors such as chronic maternal anxiety can elevate cortisol levels and trigger inflammatory responses which might subsequently disrupt foetal brain development. Concurrently, iron deficiency during critical windows of gestation can hinder the formation of brain structures and neurotransmitter systems vital for emotional regulation and cognitive function after birth. Iron deficiency and exposure to stress are among the most prevalent nutritional and environmental challenges during pregnancy, and their combined influence may substantially increase the risk of neuropsychiatric disorders in the offspring. Although the individual effects of each factor are relatively well understood, their interaction during gestation remains unexplored.

**Methods:**

In the present study, we employed human placental samples from mildly stressed and non-stressed mothers, a chronic environmental stress mouse model, and advanced *in vitro* techniques to examine whether gestational environmental stress alters placental iron transport.

**Findings:**

Our findings indicate that stress enhanced placental iron uptake and accumulation, but paradoxically reduced iron transfer to the foetus—an effect observed exclusively in females and reproducible *in vitro* following both stress exposure and dexamethasone treatment.

**Interpretation:**

These results provide insights into the sex-specific impact of environmental stress on placental and foetal iron availability and highlight a previously unrecognised pathway through which prenatal stress could influence long-term health trajectories in the offspring.

**Funding:**

This study was supported by the 10.13039/501100001711Swiss National Science Foundation (SNF grant no. 310030_197408), the 10.13039/100000001Swiss National Science Foundation via the 10.13039/501100023650National Center of Competence in Research (NCCR) TransCure, 10.13039/100009068University of Bern, Switzerland (grant no. 51NF40_185544) and the Swiss 3R Competence Centre (3RCC; grant no OC-2019-019). TF was supported by the 10.13039/100009070Hans Sigrist Foundation, Switzerland.

**Research in context**

**Evidence before this study**

A comprehensive literature search was conducted using PubMed, Google, and Web of Science. Search terms included: *iron, iron transfer, iron deficiency, placenta, stress, prenatal stress, environmental stress,* and *psychological stress*. The search covered publications from the past 35 years up to May 2025. No studies were identified that directly examined the effects of stress on placental iron allocation and transfer under environmental stress conditions.

**Added value of this study**

This study provides evidence that gestational environmental stress directly alters placental iron handling, leading to reduced foetal iron transfer despite increased placental iron uptake. By combining human placental samples, a chronic stress mouse model, and mechanistic *in vitro* approaches, the work moves beyond associative observations to identify a causal link between stress-related signalling and impaired placental iron transport. Importantly, the discovery of a female-specific vulnerability reveals a previously unrecognised sex-dependent placental response to environmental stress. These findings advance current understanding by demonstrating that prenatal stress can induce a functional foetal iron deficiency independent of maternal iron status, thereby uncovering a further biological pathway through which common environmental exposures during pregnancy may shape offspring neurodevelopment and long-term mental health risk.

**Implications of all the available evidence**

Taken together, the available evidence indicates that prenatal environmental stress can disrupt foetal iron homoeostasis through placental mechanisms that are both sex-specific and biologically consequential. While iron deficiency and maternal stress have independently been associated with adverse neurodevelopmental outcomes, the present findings suggest that their interaction during gestation may represent a previously underappreciated risk pathway. Stress-induced alterations in placental iron handling—characterised by increased placental iron uptake but impaired transfer to the foetus—may result in a functional foetal iron deficiency even in the absence of overt maternal iron depletion. This phenomenon appears to disproportionately affect female foetuses, highlighting sex-specific placental adaptations that may shape differential vulnerability to later neuropsychiatric outcomes.

These observations align with and extend prior human and animal studies linking prenatal stress exposure, dysregulated glucocorticoid signalling, and altered placental function to long-term changes in brain development and behaviour. By identifying impaired placental iron transfer as a mechanistic link between environmental stress and foetal nutrient availability, this work provides a plausible biological substrate through which prenatal stress may influence, among others, offspring mental health trajectories. Collectively, the evidence underscores the importance of considering maternal psychosocial stress alongside nutritional status in pregnancy and suggests that current strategies focused solely on maternal iron supplementation may be insufficient to fully protect foetal neurodevelopment under conditions of chronic stress.

## Introduction

Iron is a critical micronutrient that plays a pivotal role in a wide array of biological processes, including oxygen transport, DNA synthesis, enzymatic functions, myelination, and neurotransmitter synthesis in the brain.^1^ Dietary intake is the sole source of iron, which exists in two oxidation states: the ferrous (Fe^2+^) and ferric (Fe^3+^) form. Despite haem iron (ring-shaped molecular component of haemoglobin that contains Fe^2+^) comprising only about one-third of dietary iron, it exhibits significantly greater bioavailability, and accounts for up to two-thirds of the body's iron reserves. After the absorption in the intestine, Fe^2+^ is oxidised to Fe^3+^ to reach a more stable electronic configuration and to be stored in ferritin (FTH) or bound to transferrin (TF) for transport.

During pregnancy, maintaining optimal iron concentrations is essential for both maternal health and foetal development.^1^ The maternal circulation delivers iron to the placenta, where it is complexed with TF, FTH, or exists in its haem form. In general, nutrient transfer across the placenta is primarily mediated by the syncytiotrophoblasts (STB), which serve as the main barrier between the maternal and foetal circulation in both humans and rodents. The most extensively studied mechanism of placental iron trafficking involves transferrin-bound iron (TBI), which is imported via the transferrin receptor 1 (TFR1) located on the apical membrane of STB and is subsequently endocytosed into the cell.^2^^,^^3^ Once inside, iron can either be utilised or stored within the placenta, or it can be exported through ferroportin 1 (FPN1) on the basal membrane of STB, entering the foetal circulation upon crossing the foetal epithelium. While TBI is considered the predominant source of iron, other transferrin-independent iron transport mechanisms have been identified.^4^ These include the transmembrane receptor low density lipoprotein (LDL) receptor-related protein 1 (LRP1),^5^ the feline leukemia virus subgroup C receptor (FLVCR1),^6^^,^^7^ haem oxygenase (HO)-1 and HO-2,^8^^,^^9^ and–as a matter of debate–the divalent metal transporter 1 (DMT1), as reported by Chong et al.^10^

Gestational iron deficiency is a major public health concern, with a global prevalence ranging between 20% and 80% depending on the geographical location.^11^ When undiagnosed and untreated, inadequate gestational iron availability can profoundly impact both maternal and foetal health, resulting in molecular, metabolic, structural, and synaptic alterations in the offspring.^12^^,^^13^ These alterations are in turn associated with conditions such as autism, schizophrenia, and abnormal brain development. Additionally, compromised recognition memory, slower cognitive processing, and impaired bonding — effects that may persist despite postnatal iron repletion — have been observed in both humans and animals.^14^

Recent human studies have hinted that maternal prenatal stress appears to be associated with a reduction in foetal iron accrual in a sex-dependent manner.^15^^,^^16^ In turn, preclinical models of foetal iron deficiency demonstrate that critical iron-dependent processes, including monoamine neurotransmission, myelination or neuronal growth and differentiation are compromised both acutely and persistently into adulthood.^14^ Considering that both stress and iron deficiency are highly prevalent in pregnant women, investigating the impact of prenatal environmental stress on placental iron metabolism is essential for understanding the stress-related mechanisms leading to foetal programming of later life disease. Nonetheless, the placental coping mechanisms responsible for iron transport under stressful conditions remain largely unknown.

In the present study, we explored whether gestational environmental stress could influence placental iron transport. For this purpose, we utilised human tissue samples from mildly stressed and non-stressed mothers, a mouse model of chronic mild environmental stress, and sophisticated *in vitro* techniques. Altogether, we found that stress increased placental iron uptake but reduced iron transfer to the foetus exclusively in the female offspring. A similar finding was observed *in vitro* upon dexamethasone (Dex) treatment of placental cells. Our findings provide an insight into the effects of environmental stressors on iron availability *in utero* and uncover an important mechanism of foetal programming potentially predisposing male and female offspring to different later life vulnerabilities.

## Methods

### Prenatal stress protocol in mice

Swiss CD1 mice born in the animal facility of the University of Bern (Bern, Switzerland) under specific and opportunistic pathogen-free (SPOF) conditions, remained undisturbed or underwent chronic environmental stress (CES, from gestation day (GD) 1–18) while maintained in a temperature-controlled (22 ± 1 °C) vivarium. The experimental protocol was approved by the cantonal animal experimentation ordinance in Bern, Switzerland, under licence BE23/23. Females with plugs were randomly assigned to the control or CES group. The stress protocols were performed as previously described.^17^^,^^18^ Briefly, pregnant females were exposed to daily stressors, including two short manipulations during the light phase and a further overnight manipulation (n = 6 dams per group). Short manipulations included multiple cage changes, cage tilt to 30°, saturated bedding (200 mL of water), white noise (radio), no bedding in cage for 3 h and immobilisation for 30 min. Overnight manipulations included overcrowding (15 females in one cage), novel objects in the cage (glass marbles), white noise, reversed light dark cycle, illumination, cage tilt or saturated bedding.

Pregnant females (GD18) were sacrificed after an i. p. injection with a mixture containing Ketamine (20 mg/mL) and Xylazine (2 mg/mL) prepared in saline. We used 6 dams per group and one male and one female foetus per dam for the analysis. For placental and foetal weight, all females and males per litter were separately weighed and then averaged. In most cases, a 200 mg/kg pentobarbital injection was administered when the females did not fully respond to the anaesthesia and foetuses were still moving. Both the foetuses and placentas were excised, and the foetal and maternal trunk blood were collected. The sex of the foetus was determined by Sry genotyping (forward 5′-TCATGAGACTGCCAACCACAG-3′ and reverse 5′-CATGACCACCACCACCACCAA-3′). One male and one female foetus per dam were used for the mRNA, protein and iron analyses.

### Collection of healthy human placentas and isolation of primary trophoblast cells at term

50 women with full-term healthy pregnancies were enrolled at the Department of Obstetrics and Gynecology, Lindenhof Hospital Bern, Switzerland. Placental tissue samples were obtained immediately after delivery following the women's written informed consent and approval of the local ethics committee (Canton of Bern, Switzerland Basec Nr. 2016-00250). Tissue samples (from the central area, closest to the umbilical cord) were collected from the villous tree within max. 60 min after delivery, washed in Dulbecco's phosphate buffered saline (DPBS) and then immediately snap-frozen in liquid nitrogen and stored at −80 °C until RNA isolation or further processing. The sampling protocol was standardised as previously described.^19^ The exclusion criteria included maternal diabetes, hypertension, polycystic ovary syndrome, pregnancy with fibroids, and hypothyroidism/hyperthyroidism. In addition, maternal blood and blood from the foetal vein and artery of the umbilical cord were collected. We consider this cohort to be a representative sample of women in Switzerland, with about 88% of European origin, average age of 33 years at the time of maternity,^20^ and parity between 1.5 and 2 children per woman (Federal Statistical Office (FSO) or OECD, years 2015–2025).

Samples were clustered into no stress (NS) or mild stress (MS) subgroups by two step cluster analysis based on placental gene expression of the stress related genes *HSD11B2, NR3C1* or *GR, GLUT1, CRH-BP*, *HIF1a* and *FKBP5*.

Following the clustering, stress of the MS groups was confirmed by higher serum cortisol levels in the mothers and placental androgenization in the females. Primary cytotrophoblast cells (CTBs) were isolated from healthy term placenta villous tissues as previously described.^21^ Briefly, approximately 30 g of placental tissue was minced and digested for 30 min at 37 °C in saline Hanks (HBSS; mmol/L 5.37 KCl; 0.44 KH2PO4; 136.9 NaCl; 0.34 Na2HPO4; 5.55 glucose; 100 HEPES; 1.26 CaCl2; 0.81 MgSO4·7H2O; pH 7.4) plus DNase I (Sigma Aldrich, St. Louis, MI, USA) (total activity: 60.000, 40.000 and 30.000 kunitz units, respectively, for each digestion) and trypsin (Thermo Fisher Scientific, USA) (total activity: 615.000, 410.000 and 315.000 BAEE units, respectively, for each digestion). After digestion and centrifugation at 1000 RCF, the cellular pellets were resuspended in DMEM and separated by centrifugation with a Percoll gradient (Sigma Aldrich, St. Louis, MI, USA). The purity of the isolated CTBs was evaluated by staining with specific cell markers (anti-cytokeratin 7 (mean 93.4% ± 2.01 positivity), anti-vimentin (mean 2.44% ± 0.87 positivity) or anti-von Willebrand factor (mean 0.28% ± 0.06 positivity). Cells were acquired by flow cytometry (BD FACS LSRII; BD Biosciences, Franklin Lakes, NY, USA). Data acquisition for each staining was based on at least 10,000 events, performed using BD FACSDiva™ software (BD Biosciences, Franklin Lakes, NJ, USA), and analysed using FlowJo® software version 10 (FlowJo LLC, Ashland, OR, USA). Cells were then seeded at a density of 200,000 cells/cm^2^ in DMEM medium with high glucose (25 mM HEPES, 4.5 g/L glucose), supplemented with 10% foetal bovine serum and 1 × antibiotic-antimycotic (Gibco, Waltham, MA, USA) and cultured overnight to allow proper attachment (5% pCO2, 21% pO2, 74% N2).^22^

Of note, CTB cells have the capacity to spontaneously fuse over time to multinucleated STBs without the need to add stimulating factors such as forskolin to the culture medium. Cells were continuously monitored under the microscope and until fully differentiated to STBs and only then the iron uptake experiments were performed.

### Extraction and measurement of steroids from human placental tissue

The steroid measurements were carried out in collaboration with the Department of Nephrology, Kinderklinik, University Hospital, Bern. Briefly, 100 mg of chorionic tissue was homogenised on ice in 1 mL of 0.9% NaCl along with isotopically labelled steroid standards using a Polytron homogeniser (Kinematica AG, Switzerland) for 3 × 30 s. After homogenisation, 1.3 mL of methanol (GC–MS grade, Sigma, USA) along with isotopically labelled steroid standards was added to all samples. Following vortexing for about 30–60 s, samples were centrifuged at 850 RCF for 5 min at 4 °C and supernatants were transferred to fresh tubes. To the supernatants, 3 mL methanol:dicholoromethane (1:2, Sigma, Switzerland) was added and mixed on wheel for 20 min. For the liquid phase separation, all tubes were centrifuged at 850 RCF for 10 min at 4 °C. The organic phase was collected in a fresh tube and evaporated for 5 min at 56 °C with nitrogen gas. After drying, the samples were reconstituted in 200 μL of 33% methanol. Finally, placental steroid metabolites, including cortisol and corticosterone, were analysed by LC-MS (Vanquish and Orbitrap, both Thermo Fisher Scientific, Switzerland) using an in-house method developed by the Department of Nephrology, based on a published method.^23^

### Gene expression in mouse and human tissues and BeWo cells

For RNA isolation, 100 mg of placenta tissue or a confluent well of BeWo cells (b30 clone, RRID: CVCL_0044) were homogenised in TRI-Reagent (Sigma Aldrich, St. Louis, MI, USA) and then reverse transcribed using GoScript™ Reverse Transcriptase (Promega, Madison, WI, USA) following the manufacturer's instructions. Quantitative RT-PCR assays were performed using a Step One real-time PCR system (Applied Biosystems, Waltham, MA, USA) and fast SYBRGreen qPCR Master Mix (aApplied Biosystems, Waltham, MA, USA). TATA-box binding protein (Tbp), Tyrosine 3 monooxygenase/tryptophan 5-monooxygenase activation protein zeta (Ywhaz) and Glyceraldehyde 3-phosphate dehydrogenase (Gapdh) were used as reference genes. The gene expression levels iron-related and stress-related genes were quantified using the ΔCt (delta Ct) method. Details of the primers used in this study are provided in Supplementary Table S6. Validation of the cell line was done by CK7 and ZO-1 staining, and hCG secretion following forskolin treatment (Supplementary Figure S5).

### Libraries and RNAseq

Placental libraries were prepared from RNA extracted from the whole placenta, with the Illumina TruSeq Stranded Total RNA Library Preparation kit with Ribo Zero Gold (Illumina, #RS-122-2301, San Diego, CA, USA) according to the instructions, using 1000 ng total mouse placenta RNA as starting material. Libraries were quantified on a Qubit fluorometer and by qPCR with a KAPA Library Quantification Kit for Illumina libraries (#KK4828). Size distribution was checked using the Agilent High Sensitivity DNA Assay (#5067-4626) on an Agilent Bioanalyzer. Samples were denatured using 1N NaOH, diluted to a concentration of 3 nM with ExAMP master mix, and loaded onto a HiSeq 4000 machine (Illumina, San Diego, CA; #SY-401-4001) with 1% PhiX control (Illumina, #FC-110-3001, San Diego, CA, USA) spiked in. HiSeq 3000/4000 flow cells and HiSeq 3000/4000 SBS sequencing chemistry were used for paired-end sequencing with a read length of 100 bp for each direction. Sequencing was performed at the Helmholtz Center (Munich, Germany). The quality of sequencing reads was verified using FastQC 0.11.5 (http://www.bioinformatics.babraham.ac.uk/projects/fastqc). Adaptors were trimmed using cutadapt v.1.9.174 in paired-end mode. For quantification of gene expression, kallisto 0.43.175 was employed using the mouse Ensembl annotation v79 (downloaded from http://bio.math.berkeley.edu/kallisto/transcriptomes/). The 100 bootstraps sleuth 0.28.176 was used for the analysis of differentially expressed genes in gene aggregation mode, requiring q-values of <0.05 for both Wald and likelihood ratio tests, with a beta value cut-off of 0.25. For KEGG pathway analysis, GAGE 2.24.077 was used with log2 transformed, filtered and normalised TPM values extracted from sleuth. An FDR of 0.1 was applied as the cut-off for significance. Data have been deposited in the Sequence Read Archive (SRA), using the NCBI portal, under the BioProject accession number PRJNA434509 and SRA accession number SRP133035 and published in previous studies.^17^^,^^18^

### Determination of Iron, Ferritin (FTH), Transferrin (TF) and Hepcidin Content in Human Serum

Maternal and foetal venous total iron, FTH, TF in addition to foetal arterial Hepcidin were commercially analysed by LabPoint Laboratoires Médicaux SA (Avenches, Switzerland).

### Determination of total iron in mouse serum

Total serum iron quantification was performed using the Abcam Iron Assay kit (ab83366) according to the manufacturer's instructions. Foetal samples were pooled, when necessary, within litters to achieve the minimal amount (10 μL) necessary for achieving reproducible and reliable results with this kit (1–3 foetuses).

### Measurement of iron levels in human and mouse placentas

Placental concentrations of total iron were determined using a colourimetric ferrozine-based assay according to Riemer et al.^24^ For the human placenta, approximately 50 mg tissue was homogenised in 600 μL of 50 mM NaOH, then centrifuged at 1000×*g* for 10 min. The supernatant was then incubated on a shaker for 2 h. Iron standards were prepared by serial dilution of a 300 μM FeCl_3_ solution in 10 mM HCl. Iron was released from 100 μL of samples by adding 100 μL of an iron-releasing reagent, which consisted of an equal volume mixture of 1.4 M HCl and 4.5% (w/v) KMnO_4_, and incubating at 60 °C for 2 h in a fume hood.^25^^,^^26^ After cooling to room temperature, 30 μL of iron-detection reagent (containing 6.5 mM ferrozine, 6.5 mM neocuproine, 2.5 M ammonium acetate, and 1 M ascorbic acid) was added to each well and incubated for an additional 30 min at room temperature. Subsequently, 280 μL of the reaction mixture was transferred to a 96-well plate, and the absorbance at 570 nm was measured to determine the iron concentration, which was then normalised to protein concentration. For the mouse placenta, approximately 25 mg of tissue was homogenised in 300 μL 50 mM NaOH.

For the human placental explant samples, approximately 200 mg of tissue was homogenised in 3 mL hypotonic buffer and then 50 μL of the homogenate was mixed with 50 μL 100 mM NaOH. This mixture was used for the assay. The rest of the procedure from standards’ preparation to the end was the same as described above.

### Protein extraction and western blot

Human and mouse placental tissues were homogenised with a bullet blender for 1 min at 0 °C and 1100 RCF rpm (Bioprep-24R, Hangzhou Allsheng Instruments, Zhejiang, China), remixed with a 1 mL syringe, and centrifuged for 20 min at 4 °C and 14000 rpm to obtain protein extracts (Thermo Scientific, Sorvall Legend Micro 21R). Samples were extracted in hypotonic buffer (10 mM Tris–HCl pH 7.4, 10 mM NaCl, 1.5 mM MgCl_2_ and 0.1% Triton X-100) supplemented with protease inhibitor (Sigma–Aldrich, P2717-1BTL). Protein concentration was assessed by Pierce BCA protein assay kit (Thermo Scientific, cat. no. 23225). 60 μg of boiled total protein was mixed with 5× Laemmli sample buffer and separated by 8% SDS-PAGE, blotted onto nitrocellulose membranes, blocked in 5% BSA or non-fat milk for 1 h at room temperature (RT) and incubated overnight at 4 °C with primary antibodies. The different antibodies used were as follows: TFR1 (Santa Cruz Biotechnology, Sc-65882 AF647, RRID:AB_1120670, 1:500 dilution), FPN1 (Thermo Scientific, PA5-22993, RRID:AB_11154326, 1:1000 dilution), LRP1 (Cell Signalling, 26387, RRID:AB_2936897, 1:500 dilution) or β-actin antibody (Sigma–Aldrich, A2228, RRID:AB_476697, 1:5000 dilution). The secondary antibodies were incubated for 2 h at RT: Li-Cor Bioscience, IRDye680RD Goat anti-Rabbit IgG, 926-68071, 1:20000 dilution and IRDye800CW Goat anti-Mouse IgG, 926-32210, 1:20000 dilution. The membrane was analysed using a LI-COR Odyssey SA imaging system and quantified by densitometry (ImageJ^27^). Uncropped blots are shown in Supplementary Figures S2 (mouse) and 3 (human). Antibody tests are shown in Supplementary Figure S4.

For the mouse samples, 3 gels were run in the same day with the same buffers and antibodies, always in the same order: 2 control females, 2 stressed females, 2 late stress females (the latter were not included in the current manuscript), 2 control males, 2 stressed males, 2 late stress males (again the latter were not included in the current manuscript). Each band was normalised to β-Actin in the same gel, and the normalised numbers of all 3 gels were combined for each group for the statistical analysis, resulting in 6 samples per group, i.e., 24 in total. TRF1 and FPN1 were analysed in the same gel and are therefore normalised to the same β-Actin. LRP1 was run and analysed in a separate gel and has therefore a different β-Actin. Full membranes and details are shown in Supplementary Figure S2.

Similarly, the human samples were run in 2 gels and analysed similarly. Each gel was loaded in the same order starting with 3 control females, 3 stressed females, 3 control males and 3 stressed males. TRF1 and FPN1 were analysed in the same gel and are therefore normalised to the same β-Actin. LRP1 was run and analysed in a separate gel and has therefore a different β-Actin. Gels 1a and b have the same samples in the same order and the same is true for Gels 2a and b. Each band was normalised to β-Actin in the same gel, and the normalised numbers of the 2 gels were combined for each group for the statistical analysis, resulting in 6 samples per group, i.e. 24 in total. Uncropped membranes are shown in Supplementary Figure S3.

### Culture of villous explants

Immediately after delivery, pieces of cotyledons from around the central region of the placenta were dissected with a size of 2 cm^3^. Chorionic plate, maternal decidua, and areas of visible infarcts were discarded. The villous explants (approx. 800 mg explants/well) were rinsed with Mg/Ca free Hanks' Balanced Salt Solution (HBSS) and placed in Transwell® inserts (Greiner Bio One, Switzerland, 3 μm pore size) for 6-well plates containing explant culture medium for placenta (Curio Biotech, Switzerland), enriched with 2.6 mM l-glutamine. Explants (n = 11) were incubated under 21% O_2_/5% CO_2_/72% N_2_ at 37 °C in a sterile incubator. Following 24 h of incubation, the explant culture media were collected in duplicate, and stored at −20 °C until further analysis. Villous explant viability and integrity was monitored by performing an MTT test based on 3-(4,5-dimethylthiazol-2-yl)-2,5-diphenyltetrazoliumbromide (Rose Scientific, Canada), normalised to the explant weight. In addition, histological assessment of formalin fixed paraffin embedded explants was performed after H&E and Cytokeratin 7 staining. Briefly, explants were fixed in formalin (4%) for 24 h at 4 °C, transferred into a new tube with 1 mL 70% ethanol, embedded in paraffin, cut in a microtome (Cryostat Microm HM550, ThermoFisher Scientific, Waltham, MA, USA) and mounted on Superfrost Plus slides (Menzel-Glaeser, Braunschweig, Germany). The sections were rehydrated through a graded series of ethanol and H&E staining was performed. Antigen retrieval was performed in a microwave (20 min, 600 W) using preheated 10 mM sodium citrate buffer including 0.05% Tween 20 (pH 6). Finally, we routinely performed viability assays for all explant preparations (n = 9) at day 0 (approx. after 2 h in culture), day 1 (24 h) and day 2 (48 h) (Supplementary Figure S1D). The cell viability was assessed using 3-(4,5- dimethylthiazol-2-yl)-2,5-diphenyltetrazolium bromide (MTT) (Sigma–Aldrich, USA) following the instructions of the manufacturer. Absorbance measurements were carried out at 450 nm on a Vmax microplate reader (Molecular device, San Jose, CA, USA). The measurements at 24 and 48 h were normalised to values acquired in the initial culture phase (approx. after 2 h of culture) and are related to 100 mg of explant tissue. The secretion of hCG into explant culture media after 24 and 48 h was measured using a human hCG (intact) ELISA kit (RAB0092, Sigma Aldrich, St. Louis, MI, USA) following the manufacturer's instructions. The consecutive absorbance measurements were carried out at 450 nm on a Vmax microplate reader (Molecular device, San Jose, CA, USA). The concentrations of hCG released by the explants were interpolated using the respective standard curves and are related to 100 mg of tissue (n = 9).

### Proteomics

The analysis was performed by the functional genomics centre at ETH Zurich. The protein identification and quantification were performed using MaxQuant v1.6.2.3^28^ and the data were searched against the SwissProt human database. The total number of proteins with at least 2 peptides identified in all samples were 2534. A Fisher's extract Test, available in the Scaffold 5 software, was used to compare NS and MS samples, with an FDR q-value (adjusted *p* value) of 0.05 and log2 (fold change) ≤ 1.3 or ≥ 1.3. The obtained outputs were used for the generation of a QC report. The total number of unique identified peptides was 29207. Pathway enrichment analyses and visualisation were performed using Scaffold v5, Panther^29^ and String.^30^

### Iron uptake in BeWo cells following dexamethasone treatment

Cells were seeded at a density of 12,500 cells per well and cultured in low glucose DMEM medium with 10% FBS and 1× antibiotic-antimycotic for 2 days and were then treated with Dex (25 or 100 nM) or vehicle for 72 h. For the iron uptake, a mix of 0.625 mM human apo-transferrin and 7.5% NaHCO_3_ was prepared and then mixed with both cold (1 mM FeCl_3_) and hot (1 mCi/mL ^55^FeCl_3_) iron. The volume of 7.5% NaHCO_3_ is equal to the sum of the volumes of human apo-transferrin, cold and hot iron. The transferrin–iron mixture was incubated at 37 °C for 2 h to form a holo-transferrin mixture and contained a final concentration of 0.59 nM apo-transferrin, 0.27 nM ^55^FeCl_3_, and 0.9 nM FeCl_3_. After incubation, the holo-transferrin mixture was transferred to a defined volume of balanced salt solution (BSS, 136 mM NaCl, 5 mM KCl, 1 mM CaCl2, 1 mM MgCl2, 18 mM HEPES, pH 5.5) to prepare a radioactive uptake solution. This solution contained a final concentration of 87.5 nM apo-transferrin, 40.4 nM ^55^FeCl_3_, and 134.6 nM FeCl_3_. Prior to the uptake procedure, the growth medium was replaced with pre-warmed BSS and BeWo cells were incubated in this buffer to stabilise for 30 min. To start the uptake, 100 μL radioactive uptake solution was added to each well, and cells were incubated at 37 °C. Iron uptake was terminated at defined time points by washing the cells three times with cold BSS. Thereafter, 50 μL of 0.1% Triton X-100 protein lysis buffer (0.1% Triton X-100 in 5 mM Tris HCl, pH 7.4) was added to each well and a 10 μL sample from each well was taken for protein measurement. 40 μL radioactive uptake solution was added to the empty wells as dose control. Finally, 160 μL of MicroScint-20 (PerkinElmer, Germany) scintillation liquid was added to each well and the scintillation was measured using a TopCount device (PerkinElmer, Germany).

### Iron transfer in BeWo cells after dexamethasone treatment

The protocol was performed as previously described.^31^ Cells were seeded at a density of 100,000 cells/cm^2^ and cultured in low glucose DMEM medium supplemented with 10% FBS and 1× antibiotic-antimycotic in individual Transwell® inserts and incubated at 37 °C and 5% CO_2_. Following 2 days of incubation, 20 μM forskolin was added to the apical medium and cells were cultured for 3 additional days, a treatment shown to induce syncytialization and up to 80% fusion in BeWo cells.^32^^,^^33^ BeWo cells grown on Transwell® inserts adopt a well-defined apical–basal orientation, with distinct “apical” and “basal” domains.^34^^,^^35^ Four days post-seeding, cells were treated with either 25 nM Dex or vehicle for 72 h.^36^, ^37^, ^38^ For the transfer, a holo-transferrin mixture was prepared (see iron uptake assay for details), that contained a final concentration of 0.9 nM apo-transferrin, 0.9 nM ^55^FeCl_3_, and 0.9 nM FeCl_3_. After the 72 h incubation with Dex, the holo-transferrin mixture was transferred to a defined volume of BSS to prepare the radioactive transfer solution which contained a final concentration of 87.5 nM holo-transferrin, 87.5 nM ^55^FeCl_3_, and 87.5 nM FeCl_3._ The transfer was started by adding 1 mL radioactive transfer solution on the apical side and 1.2 mL phenol red-free LG-DMEM to the basal compartment. Cells were incubated at 37 °C with 5% CO_2_ and the medium was collected from the basal or apical side after 1, 6, 10 and 24 h. The entire membranes were then removed from the inserts using a scalpel and collected into scintillation vials. All samples were then treated with Irgasafe-2plus scintillation liquid and measured with a beta counter (Tri-Carb 2100 TR, Hidex Oy; Turku, Finland).

### TF-independent iron uptake in BeWo cells

The experimental procedure and calculation of the iron uptake assay were adapted from a previously published study.^39^ Briefly, BeWo cells were seeded 24 h before the beginning of the experiment into 96-well plates at a density of 60,000 cells/well in the same medium as in the previous experiments. The growth medium was removed, and the cells were washed 3 times with BSS. To measure TF-independent Fe^2+^ uptake, 100 μL of BSS (pH 5.5) supplemented with the required concentration of non-radioactive Fe^2+^ (0.1–20 μM), 1 mM ascorbic acid and 0.5 μCi/mL radioactive ^55^Fe^2+^ iron (PerkinElmer, Germany) were added into each well. The assay was performed at 37 °C for 120 min and was terminated at defined time points (30 min–2 h) by washing 3× with ice cold BSS. 10% of uptake solution volume was measured separately to obtain dosage control (DC) values. For cell lysis and ^55^Fe detection, 100 μL scintillation solution (MicroScint-20, PerkinElmer) was added to each well and the plates were shaken for 1.5 h at RT. Radioactivity was acquired by TopCount Microplate Scintillation and Luminescence Counter (PerkinElmer, Germany). Counts per minute (cpm) were determined for each timepoint and calculated as iron uptake relative to total dose using the following equation:

$$\text{Tf}-\text{independent}\;\text{Fe}\;\text{uptake}\;\left(\text{nmol}/L\right)=\frac{\text{counts}/\text{well}\left(cpm\right)\;\times \;[substrate]\left(nM\right)}{mean\;dose\;control\left(cpm\right)\;\times \;10}$$

### Iron uptake in STB cells after dexamethasone treatment

Cells were seeded in 96 well plates at a density of 200,000 cells/cm^2^ in high glucose DMEM medium supplemented with 10% foetal bovine serum and 1 × antibiotic and cultured at 37 °C and 5% CO_2_ for 66–70 h to form STBs. 25 mM or 100 nM Dex was added to the medium for 72 h. The iron uptake assay was performed as described above in the BeWo iron uptake section. The holo-transferrin mixture contained a final concentration of 0.54 nM apo-transferrin, 0.18 nM ^55^FeCl_3_, and 0.9 nM FeCl_3_. The radioactive uptake solution consisted of 87.5 nM apo-transferrin, 29.2 nM ^55^FeCl_3_, and 145.8 nM FeCl_3._ Dex treatment was started on day 3 post-seeding (when syncytial fusion *in vitro* typically plateaus^40^^,^^41^), and only after an additional 72 h of Dex exposure the uptake assay was initiated.

### Ethics

#### Human studies

Human placental tissue samples from all groups were collected after obtaining the women's written informed consent and approval of the local ethics committee (Canton of Bern, Switzerland Basec Nr. 2016-00250).

### Animal studies

The experimental procedures were performed in full compliance with animal use guidelines. The protocol was approved by the cantonal animal experimentation ordinance in Bern, Switzerland, under licence BE23/23.

### Statistical analysis

Data were expressed as mean ± standard error of the mean (SEM). Analysis performed in this work was analysed using a combination of GraphPad Prism v10 and Statistical Package for the Social Sciences (SPSS) v29.0.2.0. All statistical tests between groups were analysed using parametric or nonparametric methods following normality testing with the Shapiro–Wilk Test and are specified in each figure legend. Normally distributed data were analysed using AVOVA or MANOVA with Sidak post-hoc, otherwise data were analysed using Mann–Whitney or Kruskal–Wallis tests with Dunn's multiple comparisons tests.

Power calculations to calculate sample size were calculated based on previously published and independent data. We considered an alpha error of 0.05 and beta error (power) of 0.8. The effect sizes expected based on these calculations were large (d ≥0.8). A priori analysis was done using G∗Power 3.1 and with t test family or Mann Whitney test, difference between two independent means, two-tail evaluation, and normal parent distribution. The calculated sample size was a minimum of 6 per group. A similar approach was taken for the human cohort, with calculations based on placental gene expression from stress genes in a previous study resulting in a calculated sample size of a minimum of samples 7 per group.

### Role of funders

The funders had no role in study design, data collection, data analyses, interpretation, or writing of the report.

## Results

### Environmental stress affects placental iron homoeostasis in a sex dependent manner in mice

**Fig. 1:**
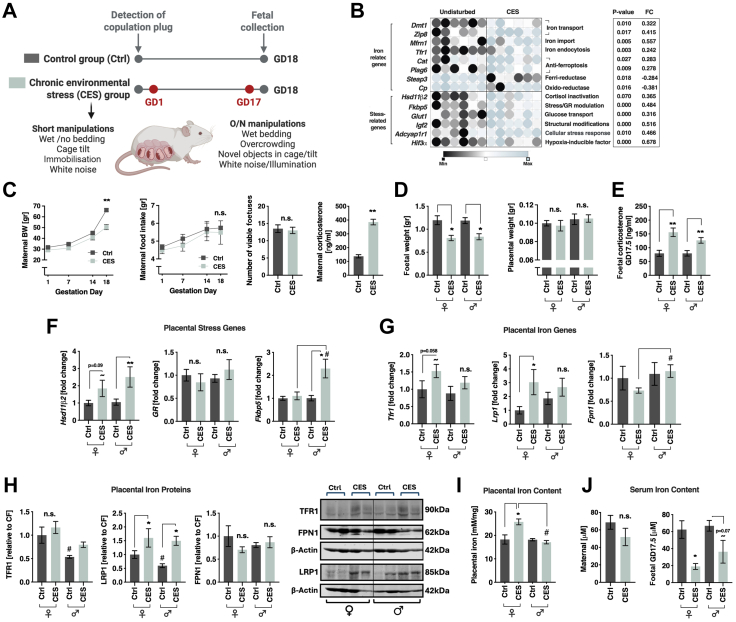
**Placental iron transport and storage was affected by chronic environmental gestational stress predominantly in female mice. (A)** Experimental design (detailed protocol see Material and Methods). **(B)** RNAseq of control and CES female placentas revealed upregulation of, among others, *Tfr1*, *Dmt1*, *Zip8* and *Mfrn1*, while *Steap3* and *Cp* were downregulated. **(C)** Dams exposed to CES weighed significantly less than controls on GD18 (Repeated measures ANOVA, F_int(3, 8)_ = 83.88, *p* < 0.001), had similar number of viable foetuses and higher levels of serum corticosterone (Mann–Whitney U = 2.88, *p* = 0.002). **(D)** Foetal weight (left) was affected by prenatal treatment (two-way ANOVA, F_(1, 12)_ = 25.66, *p* < 0.001) and was lower in the CES group. Placental weight was not affected (right). **(E)** Foetal serum corticosterone levels were significantly higher in both stress groups compared to controls (Kruskal–Wallis H = 17.03, *p* < 0.001, with Dunn's multiple comparisons test). **(F)** Placental gene expression of stress related genes was affected by the stress manipulation (MANOVA, F_(3, 18)_ = 3.61, *p* = 0.034). *Fkbp5* and *Hsd11dβ*_*2*_ were significantly increased in the male CES group (based on Sidak's post hoc). **(G)** Stress tended to influence the expression of the iron related genes (MANOVA, F_(3, 18)_ = 3.14, *p* = 0.051), specifically *Lrp1* (F_(1, 23)_ = 7.97, *p* = 0.009) and *Tfr1* (F_(1, 23)_ = 2.78, *p* = 0.035). CES females tended to have increased *Tfr1* and significantly more *Lrp1* (based on Sidak's post hoc). **(H)** Placental TFR1 protein tended to differ between the groups and was significantly lower in Ctrl males compared to Ctrl females (Kruskal–Wallis H = 7.083, *p* = 0.069, with Dunn's multiple comparisons test, left). LRP1 protein was significantly increased by stress in both sexes and was significantly lower in Ctrl males compared to Ctrl females (Kruskal–Wallis H = 14.29, *p* = 0.003, with Dunn's multiple comparisons test, centre). FPN1 protein was similar between the groups (right). **(I)** Placental iron content was significantly higher in CES females compared to Ctrl females and CES males (Kruskal–Wallis H = 11.59, *p* = 0.0089, with Dunn's multiple comparisons test). Data are presented in mean and SEM (n = 6 per group). **(J)** Serum total Iron was similar between dams of the control and CES groups (left). In contrast CES, significantly reduced total iron in the foetal serum, more robustly in females (two-way ANOVA, F_(1, 31)_ = 14.83, *p* < 0.001, differences between the groups based on Sidak's post hoc) (n = 7–9 per group). ∗*p* < 0.05, ∗∗*p* < 0.01 for differences between stress groups within sexes; #*p* < 0.05 for sex differences between the sexes within stress groups. Ctrl, Control group; CES, Chronic environmental stress. For the abbreviations and uncropped gels see supplementary Table S6.

To evaluate the effect of stress during pregnancy on placental iron availability and transfer to the foetus, dams were exposed to chronic mild environmental stress (CES) during the whole period of gestation as illustrated in Fig. 1A. RNAseq of control and CES female placentas performed in a previous study^17^ revealed upregulation of stress-related genes including 11β-Hydroxysteroid dehydrogenase 2 (*Hsd11β2*), FKBP Prolyl Isomerase 5 (*Fkbp5*), Hypoxia Inducible Factor 3 Subunit Alpha (*Hif3a*) and glucose transporter 1 (*Glut1*) (Fig. 1B, see Supplementary Table S1 for a full list of stress- and iron-related genes). Chronic mild stress further induced upregulation of the iron transporters *Dmt1*, zinc transporter 8 (*Zip8*), mitochondrial iron transporter member 37 (*Mfrn1*) and the transferrin receptor (*Tfr1*) (Fig. 1B). Interestingly, the anti-ferroptotic genes catalase (*Cat*) and phospholipase A2 group VI (*Pla2g6*) were also upregulated, hinting at placental iron buildup. In contrast, the ferri-reductase six-transmembrane epithelial antigen of prostate 3 (*Steap3*), which releases ferric-iron from TF, and the oxido-reductase Ceruloplasmin *(Cp)* were significantly reduced. This could represent a compensatory mechanism, indicating that the increased iron taken up and mobilised by the placenta may not necessarily be processed for export to the foetus. Here, we expanded our analysis to the two sexes and explored the RNAseq findings in further detail. Dams exposed to CES showed similar body weight (BW) in the first two trimesters but lower BW in the third (Fig. 1C) compared to controls, in accordance with previous studies.^17^^,^^42^ CES dams also had a similar number of viable foetuses compared to controls (Fig. 1C, centre). However, as expected, they showed higher serum corticosterone levels on gestation day (GD)18 (Fig. 1C, right). Foetal weight was reduced (Fig. 1D) and foetal corticosterone levels were increased in the CES group in both sexes (Fig. 1E). Close examination of placental expression of selected stress-related genes revealed a different response to stress among the sexes (Fig. 1F and G). Specifically, *Hsd11dβ2* and *Fkbp5* were higher in CES males compared to control males, while *Hsd11β2* only tended to be high in CES females (Fig. 1F). Analysis of the expression of genes involved in iron import and export revealed a sex-dependent pattern: we found higher *Lrp1* and a tendency to higher *Tfr1* in CES females compared to controls. On the other side, CES males had higher *Fpn1* compared to CES females (Fig. 1G). At the protein level, females presented overall higher levels of TFR1 compared to males (Fig. 1H). In addition, LRP1 was increased by CES in both sexes while FPN1 was largely unchanged (Fig. 1H). To validate what appears to be a stress-induced adaptation resulting in increased iron uptake in both sexes, we employed a ferrozine-based assay^24^ to assess the potential impact of elevated TFR1 and LRP1 expression on placental iron content across the different groups. We found that total iron concentration was higher in CES female placentas compared to both control females and CES males. Thus, the results suggest that despite different gene expression and comparable protein levels between the sexes, stress has sex-specific effects on *de facto* iron transport: placentas from CES-exposed females import and accumulate significantly more iron than those from CES-exposed males (Fig. 1I). Finally, we analysed total serum iron content in the dams and foetuses. While CES had no perceivable effects on serum iron in the pregnant females, the transport to the foetuses was significantly reduced, more robustly in females than in males (Fig. 1J).

### Gestational stress in humans increased placental iron uptake and accumulation but reduced transfer to the foetus only in females

**Fig. 2:**
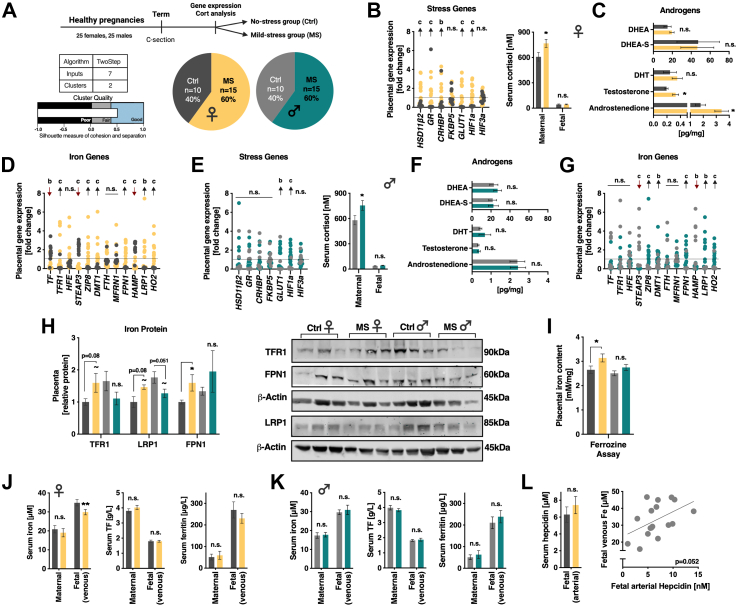
**Placental iron transport in humans was affected by gestational stress predominantly in females. (A)** Experimental design. Two step cluster analysis of placental gene expression of stress-related genes in a healthy human term cohort revealed two sub-groups that appear to have experienced different levels of prenatal stress despite being overall healthy. Accordingly, we split them into “No stress (Ctrl)” (40%) and “Mild stress (MS)” (60%) subgroups. **(B)** Placental gene expression differed between the Ctrl and the MS female groups in the stress-related genes (significance for individual genes based on Mann–Whitney tests, data presented as relative to the cohort's' average, left). Maternal serum cortisol was higher in the MS group (Mann–Whitney U = 105, *p* = 0.042), while foetal cortisol was not affected. **(C)** MS increased placental androstenedione (Mann–Whitney U = 110, *p* = 0.019) and testosterone (Mann–Whitney U = 115, *p* = 0.007) in females. **(D)** Placental gene expression differed between the Ctrl and the MS female groups in the iron-related genes (significance for individual genes based on Mann–Whitney tests, data presented as relative to the cohort's' average). **(E)** Placental gene expression differed between the Ctrl and the MS male groups in the two stress-related genes (significance for individual genes based on Mann–Whitney tests, data presented as relative to the cohort's' average, left). Similarly to the females, maternal serum cortisol was higher in the MS group (Mann–Whitney U = 101, *p* = 0.0474), while foetal cortisol was not affected. **(F)** Placental androgens did not differ between Ctrl and MS males. **(G)** Placental gene expression differed between the Ctrl and the MS male groups in the iron-related genes (significance for individual genes based on Mann–Whitney tests, data presented as relative to the cohort's' average). **(H)** At the protein level as determined by Western blotting (right panel), we found an overall significant sex × prenatal stress interaction (MANOVA, F_int(3, 18)_ = 5.622, *p* = 0.007, n = 6 per group). Placental TFR1 protein increased in MS females and decreased in MS males compared to same sex controls (F_int(1, 23)_ = 5.67, *p* = 0.027). A similar pattern was found for LRP1 (F_int(1,23)_ = 7.14, *p* = 0.015). FPN1 was significantly increased by MS only in females (F_(1, 11)_ = 5.14, *p* = 0.047). **(I)** A colourimetric ferrozine-based assay revealed iron concentrations were affected by prenatal stress, specifically in the MS female group (Kruskal–Wallis H = 9.042, *p* = 0.0287, with Dunn's multiple comparisons test). **(J)** Analysis of total iron, TF and ferritin showed similar levels in the maternal serum of both groups and sexes. Analysis of the foetal venous serum showed lower levels of total iron (one-way ANOVA, F_(1, 24)_ = 8.18, *p* = 0.009) but similar transferrin (TF) and ferritin levels in the MS female group compared to controls. **(K)** No differences in total iron, TF and ferritin were detected between control and MS males. **(L)** Arterial serum hepcidin was similar in the Ctrl and MS female group and tended to positively correlate with venous iron levels (N = 8–9). N = 10/15 per group for gene expression and serum measurements. Data presented in mean and SEM. ∗ and a: *p* < 0.05, ∗∗ and b: *p* < 0.01, c: *p* < 0.001. Black arrow upregulated in MS, red arrow downregulated in MS. For the gene abbreviations see Supplementary Table S6.

To further investigate the relevance of the mechanisms by which environmental stress affects iron transport in human placentas, we collected 50 samples from healthy term pregnancies, both female and male. They were categorised into “no-stress (NS)” and “mild stress (MS)” subgroups using a two-step cluster analysis based on the expression of selected placental stress genes (Fig. 2A), as recently published.^43^ The resulting clusters show a positive and significant correlation with maternal serum cortisol (Spearman's rho = 0.482, *p* < 0.001), based predominantly on significant positive correlations between maternal cortisol and placental DCT of *HSD11β2* (Spearman's rho = −0.346, *p* = 0.014), the glucocorticoid receptor (*NR3C1* or GR) (Spearman's rho = −0.391, *p* = 0.005) and *GLUT1* (Spearman's rho = −0.306, *p* = 0.030). The subgroups shared similar demographic characteristics, except for the higher placental weight in the male MS group (Table 1). Based on this analysis, 60% of the newborns were designated to the MS groups in both sexes (Fig. 2A). Placentas of MS females showed increased expression of *GLUT1*, in addition to the stress-related genes *HSD11β2*, corticotropin-releasing hormone binding protein (*CRH-BP*), GR, hypoxia-inducible factor 1-alpha (*HIF1a*) and *FKBP5* (Fig. 2B, left). Maternal serum cortisol was higher in the MS compared to the NS group, while the foetal cortisol, measured in the umbilical cord vein, was not affected (Fig. 2B, right). Stress during gestation is well known to produce a masculinising effect that particularly affects females, as detailed in several animal models and species.^18^^,^^44^, ^45^, ^46^, ^47^, ^48^ Thus, we next focused on this aspect to further confirm the suspected stressful prenatal environment in the MS group. We found a significant increase in placental testosterone and androstenedione (the major classic androgen in the placenta^23^) in the MS females, hinting at prenatal androgen exposure resulting from stress in this sex (Fig. 2C & F). Analysis of gene expression of iron-related genes showed differences between the groups, with increased levels of *TFR1*, *LRP1*, *DMT1*, *ZIP8*, *HO2* and *FPN1*, alongside reduced expression of *TF*, *STEAP3* and hepcidin (*HAMP*), suggesting enhanced placental iron uptake and altered iron handling in stressed conditions (Fig. 2D). The effects of stress were less pronounced in the males, and placental gene expression was significantly increased only for *GLUT1* and *HIF1a* (Fig. 2E, left). Similarly to the female group, maternal serum cortisol was higher, and foetal cortisol was not altered (Fig. 2E, right).

**Table 1:** Demographic characteristics of human cohort.

Parameter	Control females	MS females	Control males	MS males
Placental weight [g]	655 ± 35	650 ± 30	578 ± 47	721 ± 32∗
Newborn weight [kg]	3.21 ± 0.12	3.34 ± 0.06	3.34 ± 0.16	3.54 ± 0.15
Maternal Age [y]	33 ± 1.8	33 ± 1.1	33 ± 0.6	34.5 ± 1
Gestational age [d]	270 ± 0.7	271 ± 1	271 ± 0.8	272 ± 2
Gravidity	2.4 ± 0.9	2.33 ± 0.3	1.8 ± 0.4	2.14 ± 0.3
Parity	2.20 ± 0.2	1.73 ± 0.2	1.5 ± 0.4	1.8 ± 0.2
Maternal BW [kg]	66 ± 4.4	67 ± 2.8	69 ± 1	60.3 ± 2

The subgroups shared similar demographic characteristics, except for the higher placental weight in the male MS group compared to the control male group (Sex × prenatal group interaction: F_int(1, 49)_ = 6.66, *p* = 0.013, significance between the groups based on Sidak's post hoc test). N = 10–15 per group. ∗*p* < 0.05. For this reason, data analyses in Fig. 1 were stratified by sex.

Male MS placentas showed a similar trend compared to the females in the expression of the iron genes, with increased mRNA levels of *LRP1*, *DMT1*, *ZIP8*, *HO2* and *FPN1*, and reduced gene expression of *STEAP3* and *HAMP*. In contrast to the females, *TFR1* was not significantly increased (Fig. 2G). We next assessed the protein levels of TFR1, LRP1 and FPN1 to further emphasise our focus on iron importers and exporters that may influence foetal iron availability. Within the control groups, we observed higher levels of TFR1, LRP1 and FPN1 in the male placentas compared to female placentas, contrary to our findings in mice. In addition, we found a stress-induced increase in TFR1 and LRP1 in the females but a surprising decrease in the males, and enhanced FPN1 expression in MS female placentas (Fig. 2H), hinting at higher import and export in this sex. Analysis of total iron concentrations revealed higher content in placentas of MS females, suggesting they indeed imported more iron than controls (both male and female) (Fig. 2I). Finally, analysis of total iron, TF and FTH in the maternal and foetal serum revealed comparable levels in the mothers of both groups and sexes but surprisingly significantly less total iron in the serum of MS newborn females (Fig. 2J and K), despite the upregulation of FPN1 protein. To investigate the apparent discrepancy between a placental profile of iron importers and exporters that might reflect increased iron transport but in fact lower iron levels in the umbilical cord vein of female foetuses, we further analysed foetal arterial hepcidin levels in this sex, to get a hint at the foetal (liver) production. Unfortunately, we only succeeded with the collection of foetal arterial blood in a subset of the samples (n = 18), and we found no differences between the groups. Interestingly, we found a positive correlation between foetal iron levels and arterial hepcidin (Fig. 2L). While some studies suggest that placental FPN1 is regulated by foetal hepcidin,^49^ and others point at maternal hepcidin,^50^^,^^51^ more recent studies suggest that hepcidin secreted by the foetal liver does not influence placental iron transfer to the foetus but rather inhibits iron efflux from foetal hepatocytes into the foetal blood.^52^^,^^53^ Thus, while maternal stress appears to increase iron uptake and accumulation in the female placenta, a parallel compensatory mechanism by the foetus to prevent a potentially harmful iron overload^54^ may ultimately result in less available circulating iron for the female foetuses.

### Explants cultured from human stressed placental tissues show significant enrichment in pathways involved in oxygen and haem transport

**Fig. 3:**
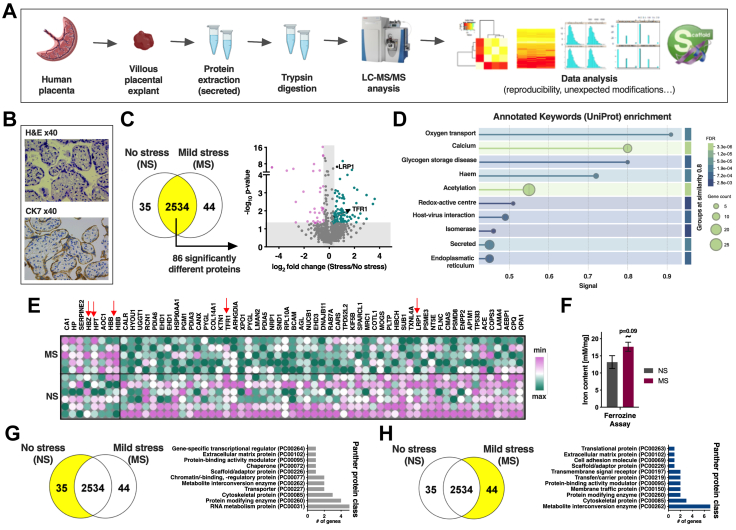
**Placental explants cultured from human term samples of mildly stressed pregnancies secrete significantly different iron-related proteins compared to non-stressed controls. (A)** Experimental design. **(B)** Hematoxylin/eosin (H&E) and Cytokeratin 7 (CK7) staining of the explants. Syncytiotrophoblasts as well as villous cytotrophoblasts are shown in brown. **(C)** Proteomics analysis of the secretion of NS and MS explants revealed 2534 proteins secreted by both groups. Among them, 86 were significantly different between the groups, including lower haemoglobin variants and higher TFR1/LRP1. **(D)** Functional enrichment analysis of the significant proteins revealed UniProt pathway enrichment for oxygen transport (KW-0561) and haem processes (KW-0349). **(E)** Heatmap of specific proteins significantly affected by mild stress and those involved in iron transport (red arrows). **(F)** Iron content measurements in explant homogenates revealed a tendency to increased iron in the MS group (*p* = 0.09). **(G)** NS explants secreted 35 exclusive proteins predominantly of the RNA metabolism class (PC00031). **(H)** MS explants secreted 44 exclusive proteins, predominantly belonging to the metabolite interconversion enzyme class (PC00262). n = 5, 6 per group. For the protein abbreviations see Supplementary Table S2. NS: No stress, MS: Mild stress.

Next, to directly assess the placenta's capacity to secrete iron homoeostasis-related factors under stress conditions, we cultured placental villous explants collected immediately after the caesarean section to closely mimic *in vivo* conditions. Placental gene expression of selected relevant markers and maternal serum cortisol were assessed to determine whether the sample belonged to the no stress control (NS) or mild stress (MS) groups (Supplementary Figure S1). Explants were cultured for 24 h after which the secreted proteins were collected, and a proteomics analysis was conducted (Fig. 3A). Hematoxylin/eosin (H&E) and Cytokeratin 7 (CK7) stainings were used to assess tissue integrity and stain the syncytiotrophoblasts as well as villous cytotrophoblasts (Fig. 3B). Viability was confirmed with a MTT test and human chorion gonadotropin (hCG) release was confirmed after 24 and 48 h (Supplementary Fig. S1C and D). Overall, the explants secreted 2534 detectable proteins (from 29207 peptides, False Discovery Rate (FDR) 1%) among which 86 were detected in all samples and were significantly different between the NS and MS groups (FDR q-value 0.05) (Fig. 3C, Supplementary Table S2). Significant enrichment pathways were identified among these proteins, remarkably for oxygen transport (KW-0561) and haem (KW-0349) (UniProt enrichment) among others (Fig. 3D).^30^ The full list of enriched molecular and biological pathways is presented in Supplementary Table S3. Specifically, we detected lower levels of haemoglobin subunits and significantly higher levels of TFR1 and LRP1 in MS explants (Fig. 3E). Since high TFR1 and LRP1 were also detected in the MS female tissues (Fig. 2C) and low haemoglobin frequently accompanies iron deficiency,^55^ our findings may further reflect higher iron uptake and lower iron transfer from the placenta to the foetus under MS conditions. Analysis of iron content in homogenates of these explants revealed a trend toward higher iron concentrations in the MS group (Fig. 3F), mirroring the pattern observed in both mouse and human placentas. Finally, MS explants secreted 44 proteins that were not detected in the NS explants (Fig. 3G, full list is shown in Supplementary Table S4), while NS explants released 35 proteins that were not found in the MS explants (Fig. 3H, the full list is shown in Supplementary Table S5). The most important classes identified among these proteins were metabolite interconversion enzymes (PC00262) in the MS group and RNA metabolism proteins in NS explants.^29^ Of particular interest in the MS group were neuromodulin, an axonal growth-associated protein^56^ and LRP2. Together with LRP1, LRP2 seems to regulate neural progenitor cells, contribute to adult brain neurogenesis and act as important cell signalling mediators to modulate neuronal growth and repair.^57^ Thus, LRP1, LRP2 and Neuromodulin may represent further important candidate proteins for foetal programming of long-term phenotype in the offspring exposed to stress. We did not find any further subgroup differences likely due to the relatively small number of samples in each group.

### BeWo choriocarcinoma cells increase iron uptake but reduce transfer when exposed to dexamethasone

**Fig. 4:**
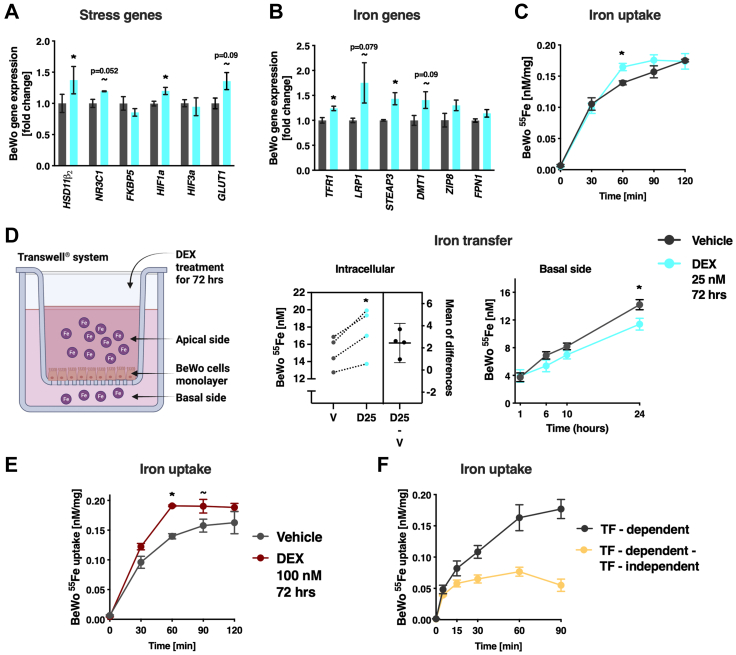
**Exposure to dexamethasone increased the expression of iron transporters and induced iron accumulation in BeWo cells. (A–C)** BeWo cells exposed to 25 nM Dexamethasone (DEX) for 72 h showed increased expression of stress-related genes (MANOVA, F_(4, 1)_ = 694.32, *p* = 0.028, n = 6) **(A)** and a similar tendency in the iron-related genes (F_(4, 1)_ = 164.4, *p* = 0.058, n = 6) **(B)**, which resulted in an increased capacity to take up iron at 60 min (significance based on ANOVA with Sidak's post hoc, n = 3) **(C)**. **(D)** The iron transfer assay revealed that exposure to DEX 25 nM for 72 h increased the intracellular iron accumulation (paired t-test, t_(3)_ = 4.38, *p* = 0.022), while transfer to the basal side was reduced (Repeated measures ANOVA, F_int(3,18)_ = 3.03, *p* = 0.063, significance based on Sidak's multiple comparisons test) (n = 4). **(E)** Treatment of BeWo cells with 100 nM DEX for 72 h resulted in a robust increase in the capacity to take-up iron (F_(1, 4)_ = 33.66, *p* = 0.004, n = 3, ANOVA with Sidak's post hoc). **(F)** The transferrin (TF)–dependent iron uptake significantly differed from the subtracted (TF–dependent minus TF-independent) iron uptake, suggesting that up to 53% of the iron was taken up by a TF–independent mechanism within the first hour (F_(1, 4)_ = 16.54, *p* = 0.015, n = 3, ANOVA with Sidak's post hoc). Data shown in means and SEM. For the gene abbreviations see Supplementary Table S6.

To study the direct effect of stress on the iron uptake and transfer capacity of placental cells, we next established iron uptake and transfer assays using the BeWo choriocarcinoma cell line exposed to a moderate dose of dexamethasone (DEX 25 nM) for 72 h to mimic a “mild stress” effect.^38^ Exposing these cells to DEX increased the expression of selected stress genes (Fig. 4A) and iron transporters (Fig. 4B) similarly to the effects observed in the mouse and human placenta. When treated with DEX, BeWo cells imported significantly more iron within 1 h of the uptake assay (Fig. 4C). When cultured in a Transwell® system, designed to mimic the maternal and foetal compartments and examine the placental transfer capacity, DEX-treated BeWo accumulated more iron intracellularly but curiously transferred less iron to the basal (foetal) compartment (Fig. 4D), similarly to our findings in female human placentas (Fig. 2G). We next increased the dose of DEX from 25 nM to 100 nM for 72 h to test whether a “stronger stressor” would have a different effect on iron transport. Indeed, exposing BeWo cells to the higher DEX dose resulted in a robust increase in the capacity to take up iron, indicating that exposure to glucocorticoids/stress affects iron homoeostasis (Fig. 4E). Finally, given the increase in LRP1 observed both in the human and mouse placental samples, in the protein secretion in the MS explants, and in the BeWo cells exposed to 25 nM DEX, we further tested the capacity of TF independent iron uptake with a protocol adapted from Pujol-Gimenez et al.^39^ Remarkably, we found that TF-independent iron uptake represented up to 50% of the iron absorbed after 1 h in BeWo cells, providing a potential alternative iron uptake mechanism that may be predominant in stress conditions (Fig. 4F).

### Female syncytiotrophoblasts import more iron than male syncytiotrophoblasts when exposed to dexamethasone

**Fig. 5:**
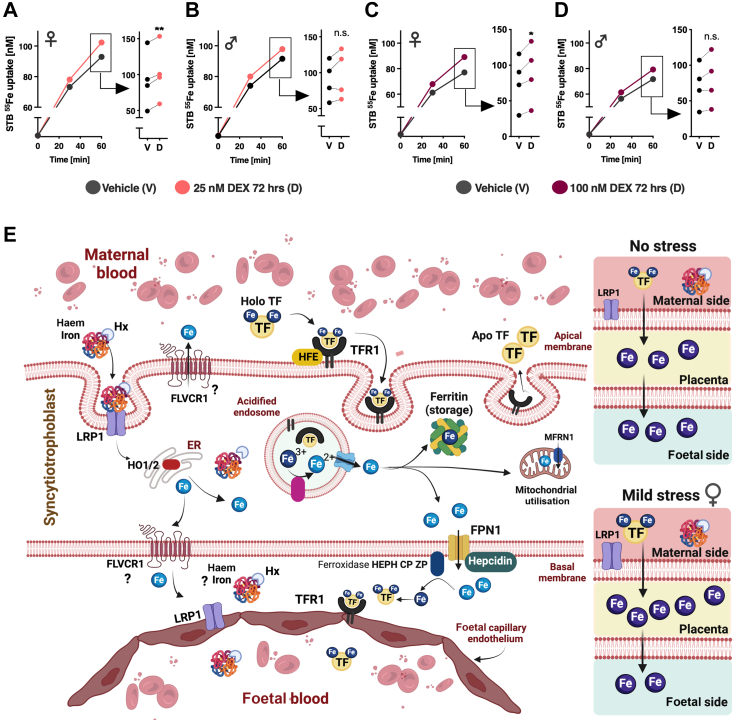
**Female syncytiotrophoblasts (STB) cells exposed to dexamethasone (DEX) present increased iron-uptake capacity after 60 min compared to male cells. (A)** Female STBs exposed to 25 nM DEX took up more iron than vehicle-treated cells after 60 min (paired t-test, t_(3)_ = 10.26, *p* = 0.002). **(B)** This effect was not significant in male STB cells. **(C)** Female STBs exposed to 100 nM DEX took up more iron than vehicle treated cells after 60 min (paired t-test, t_(3)_ = 4.19, *p* = 0.0025). **(D)** Again, this effect was not significant in male STB cells. n = 4 in all groups. **(E)** Summary figure of iron transfer mechanisms in the human placenta (biorender.com). Under mild stress conditions total iron accumulates in the female placenta and is not transferred to the foetus (right). CP: Ceruloplasmin; FLVCR1: feline leukemia virus subgroup C receptor 1, FPN1: Ferroportin 1; HEPH: Hephaestin; HFE: Human homeostatic iron regulator protein; HO-1 and HO-2: Haem oxygenase 1 and 2; Hx: Hemopexin; LRP1: Low density lipoprotein (LDL) receptor-related protein 1; MFRN1: Mitoferrin-1; TF: Transferrin; TFR1: Transferrin receptor 1; ZP: Zyklopen.

Finally, to further confirm the sex differences in placental iron transfer capacity under stress conditions, we performed a ^55^Fe uptake assay comparing syncytiotrophoblasts (STBs) isolated from female and male placentas, which were both exposed to 25 nM DEX (Fig. 5A and B) and 100 nM DEX (Fig. 5C and D) for 72 h. We found that female STBs treated with DEX in both concentrations imported significantly more ^55^Fe after 60 min compared to vehicle treated STBs (Fig. 5A and C). In males, despite a similar trend, the effects were not significant (Fig. 5B and D). Thus, consistent with previous experiments described in this manuscript, we found that the female placenta appears to require an increased supply of iron when exposed to stress (mechanism summarised in Fig. 5E).

## Discussion

Environmental or psychological distress and iron deficiency are highly prevalent and often occur independently. Iron deficiency, the most common nutritional deficiency worldwide, is especially widespread during pregnancy. Insufficient iron availability *in utero* can disrupt various aspects of foetal development and maternal health, with potential short- and long-term consequences. Since stress hormones can influence iron metabolism by affecting iron transporters and the enzymes involved in iron storage,^58^ we analysed whether mild gestational maternal stress directly affects placental iron homoeostasis and leads to changes in iron absorption, storage, and utilisation. Our findings suggest that gestational stress exerts a strong, sex-dependent influence on how the placenta handles iron. Specifically, we found that female placentas appear to import greater amounts of iron for their own consumption and storage, by increasing TBI uptake but also by activating an alternative transport mechanism. While it is unknown whether and under which circumstances the placenta may utilise and transport iron species other than TBI,^59^ it was previously shown that even Tfr1 knockout mutants, though not viable, show erythropoiesis at ED10.5.^60^ Iron homoeostasis is highly complex, and these findings indicate that alternative placental transport sources to Tfr1 exist, at least in challenging conditions. Indeed, despite the importance of haem as a source of dietary iron, little is known about its absorption mechanism.^61^ The main candidate for haem import is LRP1, a transmembrane receptor highly expressed in the human placenta, found to be the receptor for haem and its plasma-binding protein hemopexin (Hx) and integral to systemic haem clearance.^6^^,^^62^ Its role in placental iron transport is currently an emerging area of research, and in the present study we show that LRP1 is upregulated by stress *in vivo* and *in vitro*, in humans and in mice, highlighting an available alternative placental mechanism to TBI to increase placental iron supply at least under stressful conditions. Additional candidate proteins involved in haem transport include the exporters FLVCR1/FLVCR2^6^^,^^63^ and HO1/HO2,^8^^,^^62^^,^^64^ which are also expressed in the placenta but were not found to be consistently altered by stress in the current study.

Numerous enzymes utilise iron as a co-factor for their biochemical functions and play an essential role in supporting placental health and foetal development through a variety of biochemical pathways. Some of these enzymes are involved in processes like oxygen transport, metabolic reactions, antioxidant defence and DNA metabolism, including DNA repair.^65^ Moreover, iron is a crucial co-factor for several enzymes involved in the synthesis of monoamines, such as tryptophan hydroxylase (for serotonin synthesis), tyrosine hydroxylase (for dopamine synthesis) and dopamine β-hydroxylase (converts dopamine to norepinephrine).^66^ In addition, stress — physical or psychological — can have profound effects on the placenta's ability to synthesise monoamines, since stress hormones such as cortisol can influence both iron homoeostasis and monoamine production.^67^ Many foetal organs rely on the placenta to respond to environmental challenges and to produce factors that support foetal growth; however, the brain is the most vulnerable organ to disruptions in the placental barrier and secretory functions. Indeed, many neurobehavioral disorders likely trace their origin back to pathophysiological alterations in the placenta.^68^ While the presence of specific monoamine synthesising enzymes in the placenta is still a matter of discussion,^68^^,^^69^ altered placental monoamine homoeostasis could represent a potential mechanism of foetal programming resulting from stress and alterations in iron availability.

During pregnancy, the placenta takes on the roles of several vital organs, including the lungs, kidneys, and liver, to support the developing foetus. Outside of pregnancy, it is well-established that upon stress exposure, the body's demand for nutrients markedly increases to meet elevated energy requirements. This response is well-documented across various species and biological systems.^70^ The placenta is no exception and appears to behave like other organs under stress. For example, similarly to our findings showing iron accumulation in the placenta, studies in rats have consistently shown that stress led to hepatic iron accumulation,^71^ alongside increased activity in the interleukin-6-hepcidin axis. This response results in both hepatic iron buildup and hypoferremia.^72^ Additionally, chronic variable stress in rats has been shown to induce hepatic Fe^2+^ accumulation and upregulation of TFR1.^73^ Similar effects have been observed following 7-day corticosterone injections, which also led to increased TFR1 expression and hepatic iron accumulation.^74^ In general, stress activates the hypothalamic–pituitary–adrenocortical (HPA) axis and increases proinflammatory cytokines.^58^ This HPA axis dysregulation and proinflammatory state is likely mediated by IL-6 activation of hepcidin, which may impede iron absorption and disrupt systemic iron distribution. During pregnancy, a similar pattern may be taking place, since even with adequate maternal iron intake stress can trigger the activation of comparable mechanisms leading to disrupted iron transport and distribution in both the placenta and the foetus. The potential mediator could be hepcidin—either foetal or maternal—which, by sequestering iron into storage sites such as the liver and macrophages may reduce its overall availability.^58^^,^^75^ Furthermore, the iron regulatory proteins IRP1 and IRP2, which bind to iron response elements (IREs), may influence the post-transcriptional regulation of IRE-containing genes, resulting in adaptations that enable placental tissue to retain iron for its own metabolic needs.^76^ Nonetheless, the potential contribution of additional yet unidentified mechanisms cannot be ruled out.

We can only speculate about the reasons of the differential pattern of iron handling in male and female placentas and the associated yet unknown consequences. However, these results resemble our previous findings on increased nutrient (methionine) uptake in female placentas in a variety of stressful conditions including environmental stress, hypoxia, preeclampsia and even miscarriage.^43^ Thus, the female placenta appears to show a more robust adaptation strategy to stress via increase in the uptake of nutrients beyond iron. Indeed, females tend to have a more adaptable feto-placental unit, enabling their placenta to better adjust to suboptimal conditions. This adaptability enhances the placenta's ability to compensate, for example, for impaired growth by increasing the efficiency of nutrient and oxygen transfer, potentially improving survival rates.^77^ Here, the female placenta clearly prioritised iron for its own needs potentially for maintaining mitochondrial respiration, which ultimately protected the foetus from placental dysfunction.^78^

Beyond speculations about the underlying reasons for the more pronounced iron accumulation in the female placenta under stress, our findings demonstrate that stressed female foetuses are ultimately iron deficient, despite normal serum iron levels in their mothers. Given the critical role of iron in brain development from the prenatal period through adolescence, this deficiency may adversely affect myelinogenesis and synaptogenesis in females.^79^ Similar findings were reported in a study were prenatal stress reduced cord-blood ferritin concentrations. That study presented infant sex as a strong predicting variable but unfortunately did not discuss the findings any further.^80^ Another investigation performed on prenatally stressed mothers found strong associations between stress/symptoms of psychological dysfunction, and lower cord blood ferritin specifically for girls.^16^ However, another study found lower TF saturation resulting from prenatal stress only in stressed male neonates.^15^ While diet is the ultimate source of iron, foetal iron comes not just from the maternal diet directly but also from red blood cell recycling, and changes in the maternal physiology (a result of the stress experienced) can indirectly impact placental iron availability. Altogether, these findings suggest that environmental stress — though milder than other forms such as infectious stress — may exert at least part of its programming effects on the offspring by disrupting iron homoeostasis and utilisation.

Previous research shows that sex-based differences in placental gene expression extend to many adult tissues, especially the brain,^81^ highlighting the placenta's importance in identifying new treatments for mental health risks. Our current findings align with earlier work showing that stress and pregnancy complications affect LAT1 expression, methionine uptake, and methylation in both the placenta and foetus.^43^ In the referenced study, we observed a pattern similar to that of the present work: under stress, female placentas imported more methionine than male placentas, but this amino acid was predominantly retained within the placenta for its own metabolic needs. Consequently, we observed abnormal methylation in the placenta and the foetal brain, the latter effect persisted into adulthood and produced an abnormal phenotype in the female offspring.^43^

In conclusion, we found that maternal prenatal stress was associated with higher iron uptake and accumulation both in human and mouse female placentas. Considering that prenatal stress and iron deficiency are independently prevalent and frequently comorbid, studying the foetal status beyond maternal iron levels may be critical in risk assessment for infant iron deficiency and its long-term consequences. Notably, prenatal iron supplementation would likely be ineffective in preventing long-term neurodevelopmental consequences and adult mental health issues in cases such as the ones exposed in the current study. Moreover, both iron deficiency and psychological stress independently affect similar neurodevelopmental outcomes. If maternal and infant stress, along with neuroendocrine dysregulation, alters iron prioritisation toward storage tissues (e.g. the liver) and away from critical areas such as the developing brain, then reducing stress may be a more necessary and effective intervention than increasing iron intake alone.

This study has potential limitations, including the relatively small number of available samples, and clustering of the human data based only on biological analyses and without including any subjective assessments from the pregnant women which could have given a better overview of the origins and severity of the stress experienced.

Ultimately, since sex-specific differences in the term placenta extend to important adult tissues,^81^ examining placental iron-related genes could serve as a noninvasive biomarker for prenatal psychosocial stress and to identify individuals at risk. Further investigations are needed to elucidate the differential intracellular handling of iron observed in male and female placentas to determine its implications for likely differential long-term health trajectories according to foetal sex.

## Contributors

Mariana Schroeder: Conceptualization, Data curation, Formal analysis, Investigation, Methodology, Visualisation, Writing—original draft, Writing—review & editing. Nan Yi: Methodology, Validation, Writing—review & editing. Timothee C. Furrer: Methodology, Validation. Barbara Fuenzalida: Methodology, Validation. Martin Müller: Resources. Therina du Toit: Methodology, Validation. Edgar Ontsouka: Methodology, Validation, Writing—review & editing. Christiane Albrecht: Funding acquisition, Resources, Writing—review & editing. All authors read and approved the final version of the manuscript.

In addition to Mariana Schroeder, Christiane Albrecht has accessed and verified the underlying data.

## Data sharing statement

Placental RNAseq data were published in previous studies [6, 32]. Further datasets used and/or analysed during the current study are available from the corresponding author upon reasonable request.

## Declaration of interests

None.

## References

[1] Zaugg J., Solenthaler F., Albrecht C. (2022). Materno-fetal iron transfer and the emerging role of ferroptosis pathways. Biochem Pharmacol.

[2] Bastin J., Drakesmith H., Rees M., Sargent I., Townsend A. (2006). Localisation of proteins of iron metabolism in the human placenta and liver. Br J Haematol.

[3] Cao C., Fleming M.D. (2021). Localization and kinetics of the transferrin-dependent iron transport machinery in the mouse placenta. Curr Dev Nutr.

[4] Koenig M.D., Tussing-Humphreys L., Day J., Cadwell B., Nemeth E. (2014). Hepcidin and iron homeostasis during pregnancy. Nutrients.

[5] Hvidberg V., Maniecki M.B., Jacobsen C., Højrup P., Møller H.J., Moestrup S.K. (2005). Identification of the receptor scavenging hemopexin-heme complexes. Blood.

[6] Cao C., Pressman E.K., Cooper E.M., Guillet R., Westerman M., O'Brien K.O. (2014). Placental heme receptor LRP1 correlates with the heme exporter FLVCR1 and neonatal iron status. Reproduction.

[7] Jaacks L.M., Young M.F., Essley B.V. (2011). Placental expression of the heme transporter, feline leukemia virus subgroup C receptor, is related to maternal iron status in pregnant adolescents. J Nutr.

[8] Inoue R., Irie Y., Akagi R. (2021). Role of heme oxygenase-1 in human placenta on iron supply to fetus. Placenta.

[9] Bainbridge S.A., Smith G.N. (2005). HO in pregnancy. Free Radic Biol Med.

[10] Chong W.S., Kwan P.C., Chan L.Y., Chiu P.Y., Cheung T.K., Lau T.K. (2005). Expression of divalent metal transporter 1 (DMT1) isoforms in first trimester human placenta and embryonic tissues. Hum Reprod.

[11] Breymann C. (2013). Iron deficiency Anemia in pregnancy. Expert Rev Obstet Gynecol.

[12] Scholl T.O. (2005). Iron status during pregnancy: setting the stage for mother and infant. Am J Clin Nutr.

[13] Garzon S., Cacciato P.M., Certelli C., Salvaggio C., Magliarditi M., Rizzo G. (2020). Iron deficiency anemia in pregnancy: novel approaches for an old problem. Oman Med J.

[14] Georgieff M.K. (2020). Iron deficiency in pregnancy. Am J Obstet Gynecol.

[15] Zimmermann P., Antonelli M.C., Sharma R. (2022). Prenatal stress perturbs fetal iron homeostasis in a sex specific manner. Sci Rep.

[16] Campbell R.K., Tamayo-Ortiz M., Cantoral A. (2020). Maternal prenatal psychosocial stress and prepregnancy BMI associations with fetal iron status. Curr Dev Nutr.

[17] Schroeder M., Jakovcevski M., Polacheck T. (2018). Sex dependent impact of gestational stress on predisposition to eating disorders and metabolic disease. Mol Metab.

[18] Schroeder M., Jakovcevski M., Polacheck T. (2018). Placental miR-340 mediates vulnerability to activity based anorexia in mice. Nat Commun.

[19] Huang X., Baumann M., Nikitina L. (2013). RNA degradation differentially affects quantitative mRNA measurements of endogenous reference genes in human placenta. Placenta.

[20] https://www.swissstats.bfs.admin.ch/data/webviewer/appId/ch.admin.bfs.swissstat/article/issue251415432500-03.

[21] Fuenzalida B., Cantin C., Kallol S. (2020). Cholesterol uptake and efflux are impaired in human trophoblast cells from pregnancies with maternal supraphysiological hypercholesterolemia. Sci Rep.

[22] Fuenzalida B., Kallol S., Zaugg J. (2022). Primary human trophoblasts mimic the Preeclampsia Phenotype after acute hypoxia-reoxygenation insult. Cells.

[23] Karahoda R., Du Toit T., Fuenzalida B. (2025). Landscape of steroid dynamics in pregnancy: insights from the maternal-placental-fetal unit and placental models. Mol Cell Proteomics.

[24] Riemer J., Hoepken H.H., Czerwinska H., Robinson S.R., Dringen R. (2004). Colorimetric ferrozine-based assay for the quantitation of iron in cultured cells. Anal Biochem.

[25] Scott Panter S. (1994).

[26] May M.E., Fish W.W. (1978). The uv and visible spectral properties of ferritin. Arch Biochem Biophys.

[27] Schneider C.A., Rasband W.S., Eliceiri K.W. (2012). NIH Image to ImageJ: 25 years of image analysis. Nat Methods.

[28] Cox J., Mann M. (2008). MaxQuant enables high peptide identification rates, individualized p.p.b.-range mass accuracies and proteome-wide protein quantification. Nat Biotechnol.

[29] Thomas P.D., Ebert D., Muruganujan A., Mushayahama T., Albou L.P., Mi H. (2022). PANTHER: making genome-scale phylogenetics accessible to all. Protein Sci.

[30] Szklarczyk D., Morris J.H., Cook H. (2017). The STRING database in 2017: quality-controlled protein-protein association networks, made broadly accessible. Nucleic Acids Res.

[31] Yi N., Zaugg J., Fuenzalida B., Albrecht C. (2025). Iron transfer across a functional syncytialized trophoblast monolayer. Placenta.

[32] Liu M., Hassana S., Stiles J.K. (2016). Heme-mediated apoptosis and fusion damage in BeWo trophoblast cells. Sci Rep.

[33] Orendi K., Gauster M., Moser G., Meiri H., Huppertz B. (2010). The choriocarcinoma cell line BeWo: syncytial fusion and expression of syncytium-specific proteins. Reproduction.

[34] Cerneus D.P., van der Ende A. (1991). Apical and basolateral transferrin receptors in polarized BeWo cells recycle through separate endosomes. J Cell Biol.

[35] Delidaki M., Gu M., Hein A., Vatish M., Grammatopoulos D.K. (2011). Interplay of cAMP and MAPK pathways in hCG secretion and fusogenic gene expression in a trophoblast cell line. Mol Cell Endocrinol.

[36] Huang W., Zhou J., Guo J. (2021). Dexamethasone induces an imbalanced fetal-placental-maternal bile acid circulation: involvement of placental transporters. BMC Med.

[37] He B., Zhang N., Zhao R. (2016). Dexamethasone downregulates SLC7A5 expression and promotes cell cycle arrest, autophagy and apoptosis in BeWo cells. J Cell Physiol.

[38] Lye P., Bloise E., Nadeem L., Gibb W., Lye S.J., Matthews S.G. (2018). Glucocorticoids modulate multidrug resistance transporters in the first trimester human placenta. J Cell Mol Med.

[39] Pujol-Giménez J., Hediger M.A., Gyimesi G. (2017). A novel proton transfer mechanism in the SLC11 family of divalent metal ion transporters. Sci Rep.

[40] Huang X., Lüthi M., Ontsouka E.C. (2016). Establishment of a confluent monolayer model with human primary trophoblast cells: novel insights into placental glucose transport. Mol Hum Reprod.

[41] Díaz P., Wood A.M., Sibley C.P., Greenwood S.L. (2014). Intermediate conductance Ca2+-Activated K+ channels modulate human placental trophoblast syncytialization. PLoS One.

[42] Gotlieb N., Wilsterman K., Finn S.L. (2022). Impact of chronic prenatal stress on maternal neuroendocrine function and embryo and placenta development during early-to-mid-pregnancy in mice. Front Physiol.

[43] Schroeder M., Fuenzalida B., Yi N. (2024). LAT1-dependent placental methionine uptake is a key player in fetal programming of metabolic disease. Metabolism.

[44] Barrett E.S., Swan S.H. (2015). Stress and androgen activity during fetal development. Endocrinology.

[45] Barrett E.S., Parlett L.E., Sathyanarayana S. (2013). Prenatal exposure to stressful life events is associated with masculinized anogenital distance (AGD) in female infants. Physiol Behav.

[46] Biala Y.N., Bogoch Y., Bejar C., Linial M., Weinstock M. (2011). Prenatal stress diminishes gender differences in behavior and in expression of hippocampal synaptic genes and proteins in rats. Hippocampus.

[47] Barrett E.S., Redmon J.B., Wang C., Sparks A., Swan S.H. (2014). Exposure to prenatal life events stress is associated with masculinized play behavior in girls. Neurotoxicology.

[48] Kalinichenko L.S., Smaga I., Filip M., Lenz B., Kornhuber J., Müller C.P. (2023). Sex-specific effects of different types of prenatal stress on foetal testosterone levels and NMDA expression in mice. Behav Brain Res.

[49] Ganz T. (2011). Hepcidin and iron regulation, 10 years later. Blood.

[50] Sangkhae V., Fisher A.L., Chua K.J., Ruchala P., Ganz T., Nemeth E. (2020). Maternal hepcidin determines embryo iron homeostasis in mice. Blood.

[51] Fisher A.L., Nemeth E. (2017). Iron homeostasis during pregnancy. Am J Clin Nutr.

[52] Kämmerer L., Mohammad G., Wolna M., Robbins P.A., Lakhal-Littleton S. (2020). Fetal liver hepcidin secures iron stores in utero. Blood.

[53] Ganz T. (2020). The role of hepcidin in fetal iron homeostasis. Blood.

[54] Quezada-pinedo H.G., Cassel F., Duijts L. (2021). Maternal iron status in pregnancy and child health outcomes after birth: a systematic review and meta-analysis. Nutrients.

[55] Wachsmuth N.B., Aigner T., Völzke C., Zapf J., Schmidt W.F. (2015). Monitoring recovery from iron deficiency using total hemoglobin mass. Med Sci Sports Exerc.

[56] Wijnberger L.D.E., Nikkels P.G.J., Van Dongen A.J.C.M. (2002). Expression in the placenta of neuronal markers for perinatal brain damage. Pediatr Res.

[57] Landowski L.M., Pavez M., Brown L.S. (2016). Low-density lipoprotein receptor-related proteins in a novel mechanism of axon guidance and peripheral nerve regeneration. J Biol Chem.

[58] Reid B.M., Georgieff M.K. (2023). The interaction between psychological stress and iron status on early-life neurodevelopmental outcomes. Nutrients.

[59] Sangkhae V., Nemeth E. (2019). Placental iron transport: the mechanism and regulatory circuits. Free Radic Biol Med.

[60] Levy J.E., Jin O., Fujiwara Y., Kuo F., Andrews N.C. (1999). Transferrin receptor is necessary for development of erythrocytes and the nervous system. Nat Genet.

[61] Sangkhae V., Fisher A.L., Ganz T., Nemeth E. (2023). Iron homeostasis during pregnancy: maternal, placental, and fetal regulatory mechanisms. Annu Rev Nutr.

[62] Mégier C., Peoc’h K., Puy V., Cordier A.-G. (2022). Iron metabolism in normal and pathological pregnancies and fetal consequences. Metabolites.

[63] Keel S.B., Doty R.T., Yang Z. (2008). A heme export protein is required for red blood cell differentiation and iron homeostasis. Science.

[64] Barber A., Robson S.C., Myatt L., Bulmer J.N., Lyall F. (2001). Heme oxygenase expression in human placenta and placental bed: reduced expression of placenta endothelial HO-2 in preeclampsia and fetal growth restriction. FASEB J.

[65] Puig S., Ramos-Alonso L., Romero A.M., Martínez-Pastor M.T. (2017). The elemental role of iron in DNA synthesis and repair. Metallomics.

[66] Fitzpatrick P.F. (2023). The aromatic amino acid hydroxylases: structures, catalysis, and regulation of phenylalanine hydroxylase, tyrosine hydroxylase, and tryptophan hydroxylase. Arch Biochem Biophys.

[67] Melnikova V., Lifantseva N., Voronova S., Bondarenko N. (2024). Prenatal stress modulates placental and fetal serotonin levels and determines behavior patterns in offspring of mice. Int J Mol Sci.

[68] Rosenfeld C.S. (2021). The placenta-brain-axis. J Neurosci Res.

[69] Perić M., Bečeheli I., Čičin-Šain L., Desoye G., Štefulj J. (2022). Serotonin system in the human placenta – the knowns and unknowns. Front Endocrinol (Lausanne).

[70] Lopresti A.L. (2020). The effects of psychological and environmental stress on micronutrient concentrations in the body: a review of the evidence. Adv Nutr.

[71] Zhao M., Liu L.J., Li X.G. (2014). Psychological stress leads to hepatic iron accumulation and disturbs iron homeostasis. J Chem Pharm Res.

[72] Zhao M., Chen J., Wang W. (2008). Psychological stress induces hypoferremia through the IL-6–hepcidin axis in rats. Biochem Biophys Res Commun.

[73] Jiang S., Guo T., Guo S. (2021). Chronic variable stress induces hepatic Fe(II) deposition by up-regulating ZIP14 expression via miR-181 family pathway in rats. Biology (Basel).

[74] He F., Ma L., Wang H., Shen Z., Li M. (2011). Glucocorticoid causes iron accumulation in liver by up-regulating expression of iron regulatory protein 1 gene through GR and STAT5. Cell Biochem Biophys.

[75] Ganz T. (2006). Hepcidin and its role in regulating systemic iron metabolism. Hematology.

[76] O'Brien K.O. (2022). Maternal, fetal and placental regulation of placental iron trafficking. Placenta.

[77] Meakin A.S., Cuffe J.S.M., Darby J.R.T., Morrison J.L., Clifton V.L. (2021). Let's talk about placental sex, baby: understanding mechanisms that drive female- and male-specific fetal growth and developmental outcomes. Int J Mol Sci.

[78] Parrow N.L., Fleming R.E. (2020). The selfishly selfless placenta. J Clin Invest.

[79] Nnah I.C., Wessling-Resnick M. (2018). Brain Iron homeostasis: a focus on microglial Iron. Pharmaceuticals.

[80] Armony-Sivan R., Aviner S., Cojocaru L. (2013). Prenatal maternal stress predicts cord-blood ferritin concentration. J Perinat Med.

[81] Olney K.C., Plaisier S.B., Phung T.N. (2022). Sex differences in early and term placenta are conserved in adult tissues. Biol Sex Differ.

