# Supplementary material for: Placental iron transport under maternal stress: a missing link in foetal programming and mental health
Source: eBioMedicine. 2026 Feb 13;125:106170. doi: 10.1016/j.ebiom.2026.106170 (PMC12925318; doi:10.1016/j.ebiom.2026.106170)
Supplement: Supplementary Figures [file mmc1.docx]

**Supplementary Figure 1.** Determination of cluster group in placental explants.


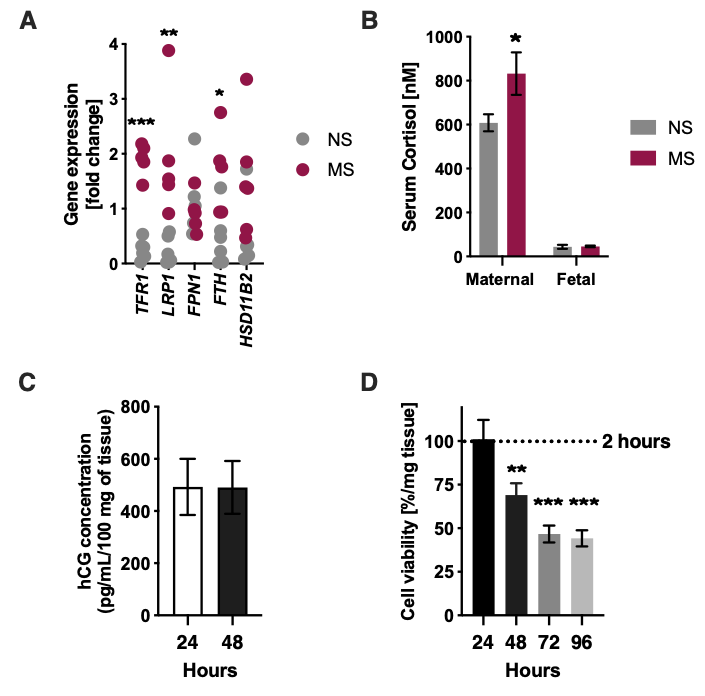


**Supplementary Figure 1. (A)** Gene expression of selected genes in placental homogenates. TRF1, LRP1 and FTH were higher in the MS group. n = 11. **(B)** Maternal and fetal serum cortisol by cluster. Maternal cortisol was higher in the MS group (F_(1,10_)=5.28, p = 0.047), while fetal cortisol was not affected. n = 5-6. (C) Secretion of hCG into explant culture media after 24 and 48 hours was similar between the groups. N = 9. (D) Cell viability test using 3-(4,5-dimethylthiazol-2-yl)-2,5-diphenyltetrazolium bromide (MTT). The measurements were normalized to values acquired in the initial culture phase (approx. after 2 hours of culture) and are related to 100 mg of explant tissue (n = 9). Cell viability was stable after 24 hours but was reduced after 48 (t_(8)_ = 4.602, p=0.0018), 72 (t_(8)_ = 10.97, p<0.001) and 96 (t_(8)_ = 11.97, p<0.001) hours.

Abbreviations: NS: no stress, MS: Mild stress. TFR1: Transferrin receptor 1; LRP1: Low-density lipoprotein receptor-related protein; FPN1: Ferroportin 1; FTH: Ferritin, Hsd11dβ2: 11β-Hydroxysteroid dehydrogenase 2.

**Supplementary Figure 2.** Uncropped Western Blot membranes.

Mouse transferrin receptor 1 (TFR1) and ferroportin 1 (FPN1) (a)


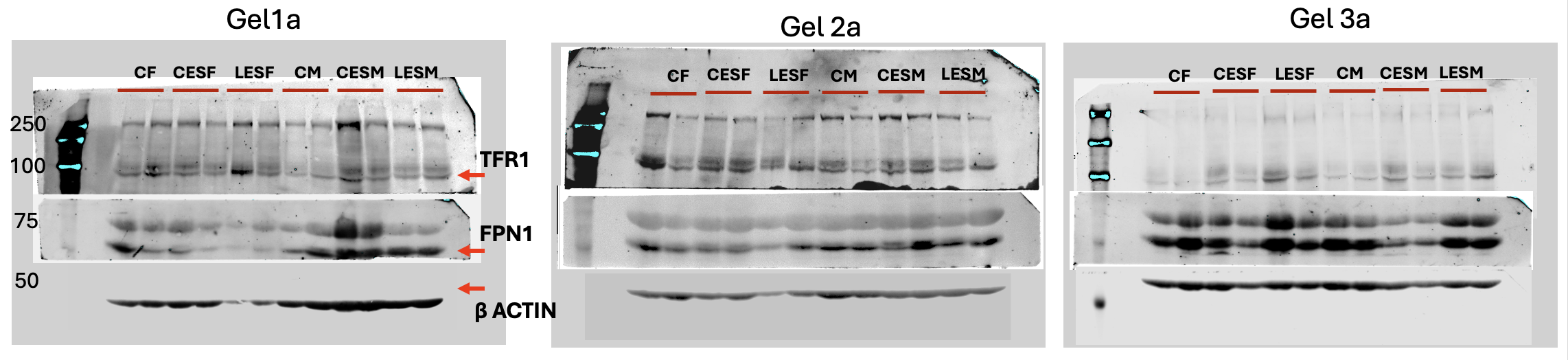


Mouse low-density lipoprotein receptor-related protein (LRP1) (b)

*
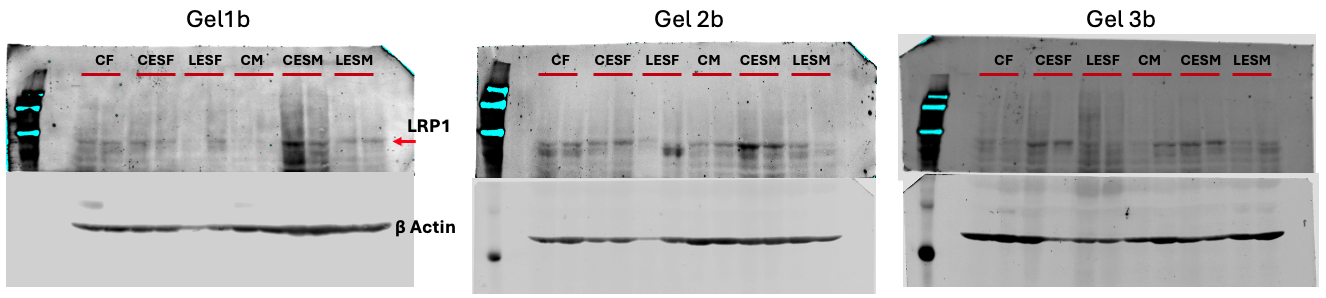
*

CF: Control female; CESF: chronic environmental stress female; CM: Control male; CESM: chronic environmental stress male. LESF and LESM are not part of the current manuscript.

Figure 1I was constructed cropping Gels 3a (TFR1 and FPN1 with ACTIN) and 3b (LRP1 with ACTIN) and removing the irrelevant (LES) groups as follows:

*
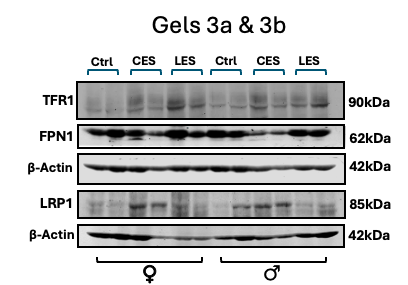
*

*
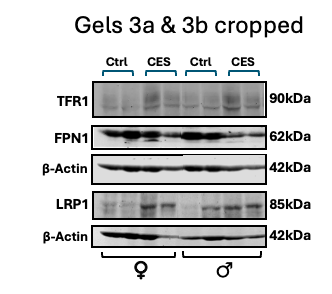
*

**Supplementary Figure 3.** Uncropped Western Blot membranes.

Human transferrin receptor 1 (TFR1) and Human ferroportin 1 (FPN1) (a)


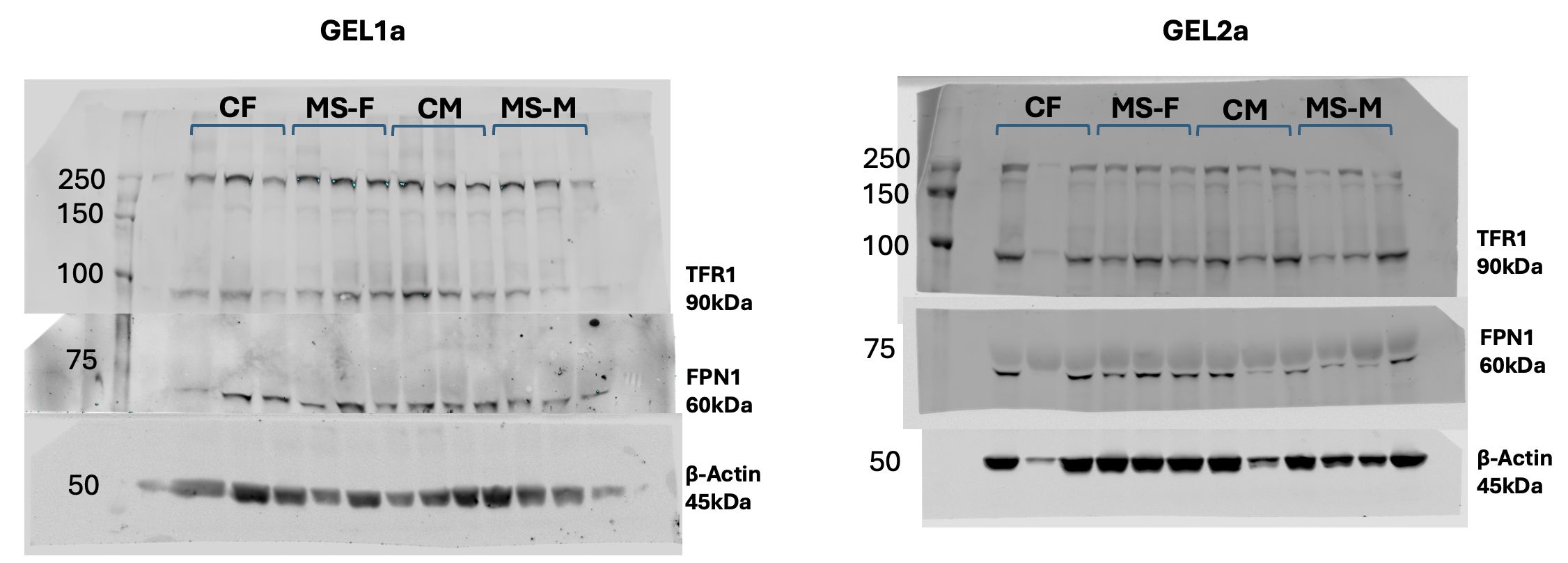


Human low-density lipoprotein receptor-related protein (LRP1) (b)


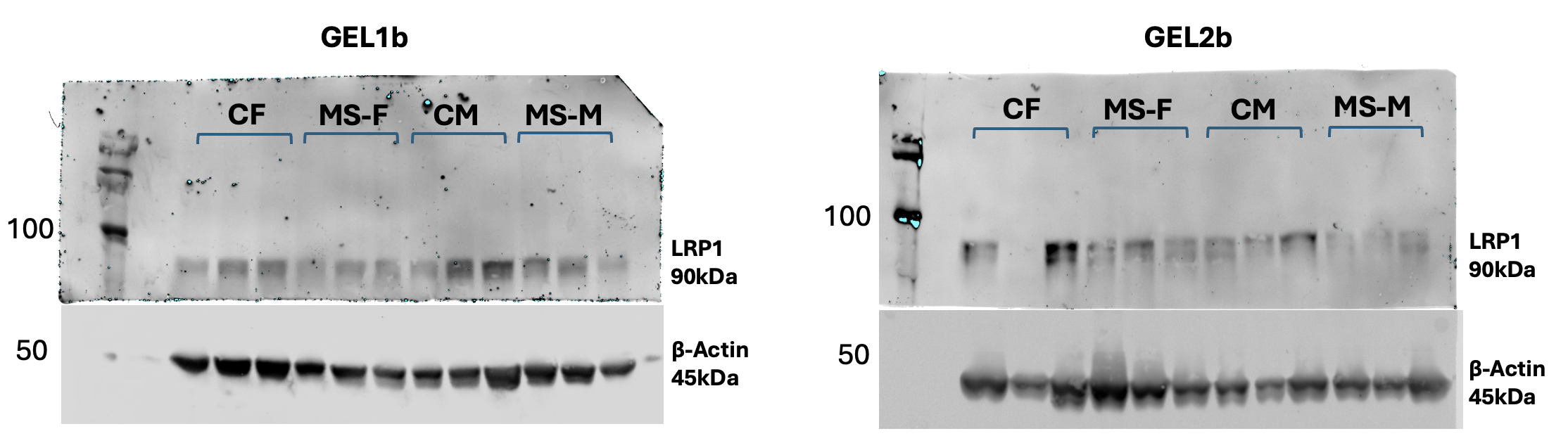


CF: Control female; MS F: Mild stress female; CM: Control male; MS M: Mild stress male.

Figure 2E was constructed cropping Gels 1a (TFR1 and FPN1 with ACTIN) and 1b (LRP1 with ACTIN) as follows:


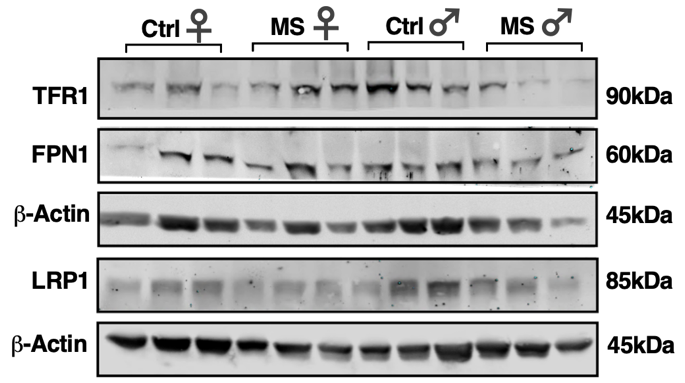


**Supplementary Figure 4.** Validation of antibodies.


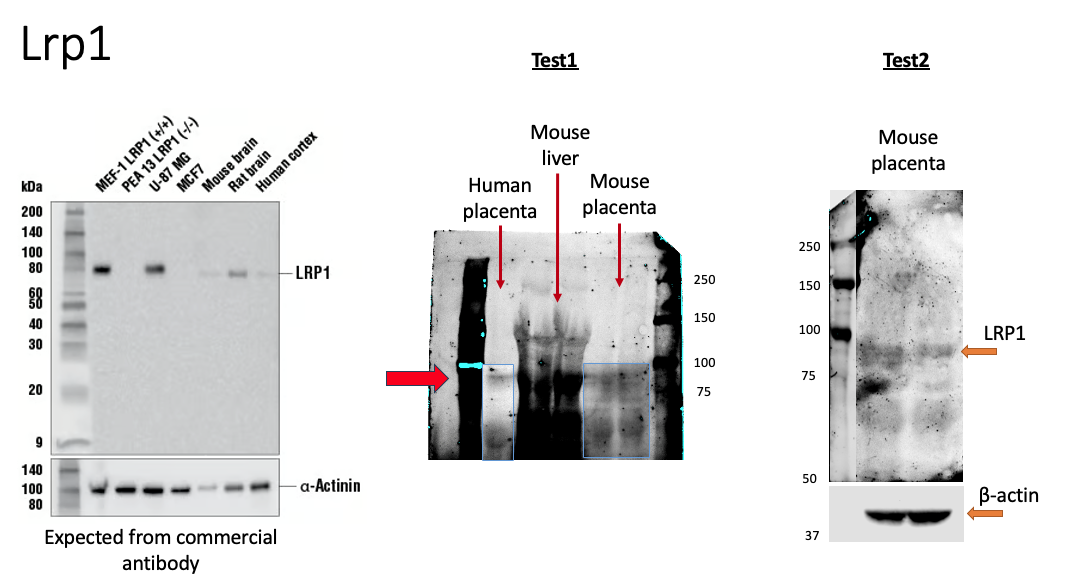


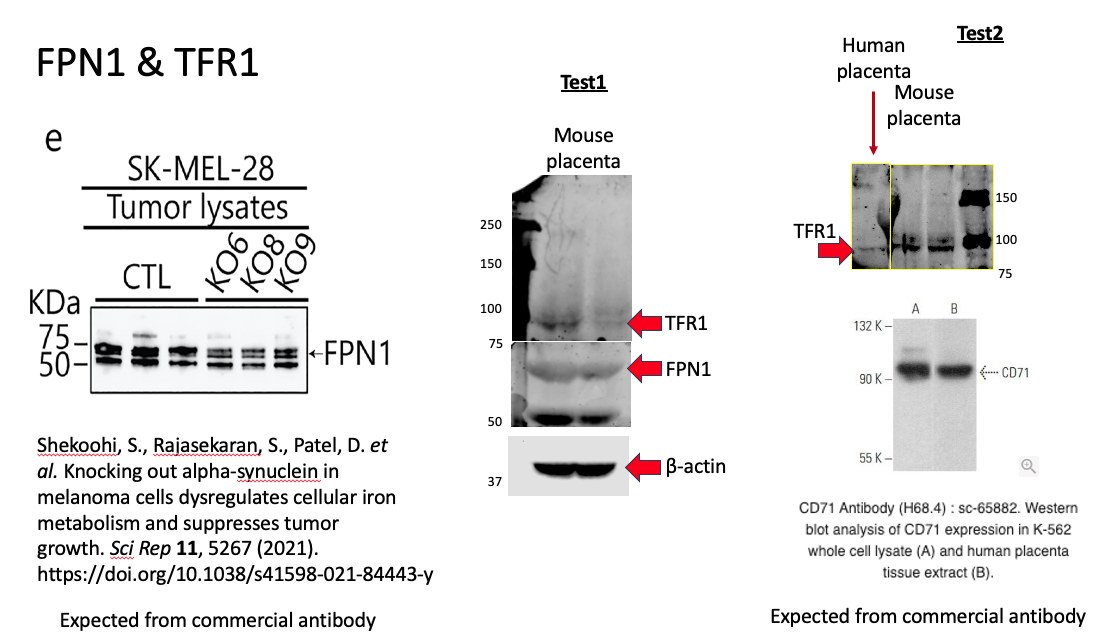


**Supplementary Figure 5.** BeWo cells validation**.**

**
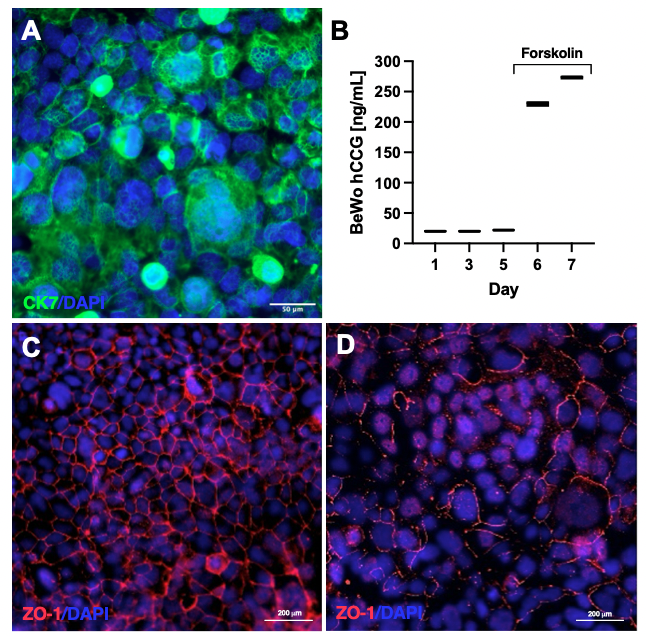
**

**Supplementary Figure 5.  (A)** Cytokeratin 7 expression in BeWo cells (CK7, 1:100, epithelial marker; Dako, Denmark, cat. no. M7018) was assessed by immunofluorescence on coverslips. Cells were fixed with 4% paraformaldehyde (PFA) and permeabilized using PBS containing 0.05% Triton X-100. An Alexa Fluor^®^ 488–conjugated goat anti-mouse (H+L) secondary antibody (λ_ex/λ_em: 495/519 nm; 1:1000 dilution, (Thermo Fisher Scientific, USA) was used. Nuclei were stained with 4′,6-diamidino-2-phenylindole dihydrochloride (DAPI; 0.1 µg/mL, blue). Immunofluorescence images show the merged signals of CK7 and DAPI. Images were acquired using a 40× objective on a Zeiss LSM 710 confocal microscope equipped with Airyscan**. (B)** hCG secretion from BeWo cells grown on Transwell^®^ inserts measured by enzyme-linked immunosorbent assay (ELISA, Sigma-Aldrich). Data presented in median and range, n=3. **(C-D)** Immunofluorescence of ZO-1 (red) and DAPI (blue) visualizing the barrier integrity and BeWo syncytialization before and after 3-days of forskolin stimulation.
